# Strain-based and sex-biased differences in adrenal and pancreatic gene expression between KK/HlJ and C57BL/6 J mice

**DOI:** 10.1186/s12864-021-07495-4

**Published:** 2021-03-12

**Authors:** Angela Inglis, Rosario Ubungen, Sarah Farooq, Princess Mata, Jennifer Thiam, Soad Saleh, Sherin Shibin, Futwan A. Al-Mohanna, Kate S. Collison

**Affiliations:** Department of Cell Biology, King Faisal Specialist Hospital & Research Centre, PO BOX 3354, Riyadh, 11211 Saudi Arabia

**Keywords:** Microarray, Strain, Gene expression, Glucose homeostasis, Insulin tolerance test, C57BL/6 J, KK/HlJ, Sex-dependent

## Abstract

**Background:**

The ever-increasing prevalence of diabetes and associated comorbidities serves to highlight the necessity of biologically relevant small-animal models to investigate its etiology, pathology and treatment. Although the C57BL/6 J model is amongst the most widely used mouse model due to its susceptibility to diet-induced obesity (DIO), there are a number of limitations namely [1] that unambiguous fasting hyperglycemia can only be achieved via dietary manipulation and/or chemical ablation of the pancreatic beta cells. [2] Heterogeneity in the obesogenic effects of hypercaloric feeding has been noted, together with sex-dependent differences, with males being more responsive. The KK mouse strain has been used to study aspects of the metabolic syndrome and prediabetes. We recently conducted a study which characterized the differences in male and female glucocentric parameters between the KK/HlJ and C57BL/6 J strains as well as diabetes-related behavioral differences (Inglis et al. 2019). In the present study, we further characterize these models by examining strain- and sex-dependent differences in pancreatic and adrenal gene expression using Affymetrix microarray together with endocrine-associated serum analysis.

**Results:**

In addition to strain-associated differences in insulin tolerance, we found significant elevations in KK/HlJ mouse serum leptin, insulin and aldosterone. Additionally, glucagon and corticosterone were elevated in female mice of both strains. Using 2-factor ANOVA and a significance level set at 0.05, we identified 10,269 pancreatic and 10,338 adrenal genes with an intensity cut-off of ≥2.0 for all 4 experimental groups. In the pancreas, gene expression upregulated in the KK/HlJ strain related to increased insulin secretory granule biofunction and pancreatic hyperplasia, whereas ontology of upregulated adrenal differentially expressed genes (DEGs) related to cell signaling and neurotransmission. We established a network of functionally related DEGs commonly upregulated in both endocrine tissues of KK/HlJ mice which included the genes coding for endocrine secretory vesicle biogenesis and regulation: PCSK2, PCSK1N, SCG5, PTPRN, CHGB and APLP1. We also identified genes with sex-biased expression common to both strains and tissues including the paternally expressed imprint gene neuronatin.

**Conclusion:**

Our novel results have further characterized the commonalities and diversities of pancreatic and adrenal gene expression between the KK/HlJ and C57BL/6 J strains as well as differences in serum markers of endocrine physiology.

**Supplementary Information:**

The online version contains supplementary material available at 10.1186/s12864-021-07495-4.

## Introduction

Type 2 diabetes (T2D) is a progressive disease with multiple contributing etiologies, in which diverse genetic backgrounds interact with environmental factors to promote insulin insufficiency, frequently associated with peripheral insulin resistance, and subsequent inability of pancreatic beta cells to compensate [1, 2]. Obesity is a major risk factor for type 2 diabetes, and the steep escalation in the global prevalence of both obesity and T2D has been accompanied by emerging evidence of clinically relevant sex-related differences, with males diagnosed at a younger age and with a lower body mass index (BMI), whilst females generally have a higher total fat mass, which is a major risk factor for T2D [3, 4]. There are also significant sex-related differences in the incidence of diabetic complications as well as differences in the counter-regulatory adrenal response to hypoglycemia and exercise stress [5–8]. Additionally, clinical studies have shown that there are a number of important sex-related differences in pancreatic islet function and pathophysiology, as well as the response to treatment regimens [9]. These realizations have emphasized the need for animal models which combine cost-effectiveness with biological relevance in order to further our understanding of the etiology and treatment of diabesity. Rodent models are particularly appropriate because they are plentiful, cost-effective and technically suitable for manipulation. They have also been used to provide detailed analysis of gene expression in relevant target organs using validated microarray analysis. A further advantage is that these models can also be used to study the etiology and treatment of diabetes-associated aspects of behavior such as anxiety, depression and cognitive impairment [10, 11].

Experimental diabetic models can be classified into (a) spontaneous or genetically-derived, (b) transgenically derived, (c) diet-induced obesity (DIO), (d) chemically induced or (e) surgically induced. In this regard the C57BL/6 J strain is especially well-suited for prediabetes research due to its susceptibility to DIO [12]. Even when placed on a standard chow diet, C57BL/6 J mice develop moderately elevated fasting blood glucose and HbA1c levels, as well as attenuated glucose tolerance compared to a number of inbred strains [1, 13, 14]. However, overt fasting hyperglycemia (fasting blood glucose levels of ≥250 mg/dL [15]) may only be achieved by dietary manipulation and/or chemical ablation of the pancreatic beta cells [1]. Additionally, heterogeneity exists in the obesogenic effects of hypercaloric feeding together with sex-dependent differences with males being more responsive [16, 17], a situation which does not accurately reflect the human condition. Notwithstanding, the C57BL/6 J DIO model has been highly successful despite the over-reliance on males within the experimental paradigms [18, 19], however several alternative strains are available which encompass various advantages and differences (1). One of these, the KK inbred strain, has been utilized for the investigation of the metabolic syndrome and prediabetes due to their inherent glucose intolerance, insulin resistance and hyperinsulinemia [20]. Initially bred for increased body weight, KK mice are characterized by moderate hyperglycemia and hyperphagia even when maintained on a standard chow diet [21]. The selectively-bred KK/HlJ substrain [22] has been used in the study of diabetic nephropathy [23, 24], fatty liver disease [25] and corneal degeneration [26]. Fewer studies have addressed sex-dependent differences within this strain, and even less is known about their suitability for behavioral research.

We recently performed a study in which we compared the glucose- and insulin-related physiological characteristics of both male and female KK/HlJ mice with those of the well-characterized C57BL/6 J strain, as well as strain- and sex-dependent differences in diabetes-related behavioral characteristics [27]. Other reports have demonstrated that in addition to their susceptibility to diabesity and as part of the counter-regulatory response to hypoglycemia, the KK/HlJ strain is prone to albuminuria and age-related vascular mineralization [28]. Morphological analysis of KK mice pancreatic Islets have revealed marked hypertrophy and hyperplasia, with enlargement of the Golgi and Endoplasmic Reticulum [29]. Additionally KK mice display enlargement of the adrenal cortex with hyperplasia of the zona fasciculate and reticularis cells, a more extensive Golgi apparatus and markedly fewer lipid vesicles present [30]. In order to characterize these differences to further our understanding of KK/HlJ glucocentric metabolism, we have now employed microarray genomic analysis [31]. The aim of the present study was therefore to examine the strain- and sex-related differences in pancreatic and adrenal gene expression profiles which might account for the glucose- and insulin-related physiological differences and similarities between the C57BL/6 J and KK/HlJ strains. It is hoped that the investigation will contribute to the future development of biological sex-based and personalized therapies.

## Materials and methods

### Animals and treatments

C57BL/6 J (stock #000664) and KK/HlJ (stock #002106) mice of both sexes were obtained from the Jackson Laboratory (Maine, USA) in two batches aged between 5 and 12 weeks upon arrival, and acclimatized for between 7 and 20 days in a sterile holding facility with veterinary monitoring before transfer to the main animal facility, where they were bred to increase study numbers. At the holding facility all animals were given free access to standard chow (Saudi Grains Organization (SAGO) Riyadh, catalog #1005: 4% crude fat, 20% crude protein, 3.5% crude fiber) and ad libitum water as previously described [27]. All next generation animals were used for the experiments described below. Experimental subjects were housed 3 to a cage (*N* = 18 per strain and per sex), in a controlled environment (pathogen-free conditions of 12 h light/dark cycle, 22 ± 2 °C) with free access to standard chow and water. Food and water intake was measured at 6 and 13 weeks of age by the subtraction method. The care of the animals was in accordance with the protocols approved by the Animal Care and Use Committee of the King Faisal Specialist Hospital & Research Centre.

### Glucocentric measurements

#### Insulin tolerance test (ITT)

Changes in the response to exogenous insulin challenge were assessed by a random-fed ITT performed at 18 weeks of age. A baseline blood glucose reading was established from arterial blood collected from the tail using a glucometer (Contour Next, Bayer NJ). An intraperitoneal injection of insulin (Sigma, IL) was administered at a dose of 0.75 U/kg body weight, and whole blood glucose levels were measured at 15, 30, 45 and 60 min after injection as previously described [27]. Assessment of insulin tolerance was made after calculating the Area Under the Curve for glucose (AUC _GLUCOSE_), the rate of glucose utilization (K ^ITT^), and the half-life of glucose levels (T ^1/2^). AUCs were calculated using the trapezoidal rule. K ^ITT^, defined as the percentage decline in glucose per minute, was calculated from the natural log (Ln) of glucose concentrations between time t1 and t2, formula K ^ITT^ = (Ln(t1) − Ln(t2))t2 − t1 × 100. The serum T^1/2^, defined as the time in minutes required for the glucose concentration to be halved, was calculated as [32]:

$$ {\mathrm{T}}^{1/2}=0.693\;{\mathrm{K}}^{\mathrm{ITT}}\times 100. $$

#### Biochemical analysis

At the conclusion of the study (20 weeks of age), blood glucose levels were assessed in 6-h fasted animals using arterial blood collected from the tail [33]. Mice were then euthanized with a mixture of xylazine and ketamine (10 mg/kg and 100 mg/kg respectively) and blood was rapidly collected from the inferior vena cava and processed for further analysis. Plasma insulin was measured by ELISA (Cat# 90080; Crystal Chem Inc., IL). The homeostasis model assessment of insulin resistance (HOMA-IR) was calculated from fasted insulin and glucose levels according to the formula [34]:

$$ \mathrm{HOMA}\ \mathrm{IR}=\mathrm{serum}\ \mathrm{insulin}\ \left(\mathrm{mmol}/\mathrm{L}\right)\ast \Big(\mathrm{blood}\ \mathrm{glucose}\ \left(\mathrm{mmol}/\mathrm{L}\right)/\mathrm{22.5.} $$

Serum glucagon, corticosterone and aldosterone analysis was performed using Crystal Chem Cat# 81518, Enzo Life Sciences kit Cat# ADI-900-097 and Enzo Life Sciences kit Cat# ADI-901-173 respectively, according to manufacturer’s recommendations. ELISAs were used to detect changes in the metabolic hormones Leptin and C-peptide, as well as cytokines IL-6 and TNF alpha according to manufacturers’ instructions (Mouse Metabolic Magnetic Bead Multiplex assay, Catalog #MMHMAG-44 K; MerkMillipore).

### RNA isolation

Total RNA was prepared from snap-frozen male and female adrenal and pancreatic tissue using Qiagen RNeasy Lipid Tissue Mini Kit Cat # 74804 (Qiagen, CA, USA) according to the manufacturer’s instructions, and stored at − 80 ^o^ C, as described previously [35]. This method was slightly modified for pancreatic RNA extraction, according to De Lisle, 2014 [36]. RNA integrity was measured using a 2100 Bioanalyzer instrument and an RNA 6000 Nano LabChip assay (Agilent Technologies, CA, USA). RNA concentrations were determined by absorption at 260-nm wavelength with an ND-8000 spectrophotometer (Nanodrop Technologies, DE, USA).

### Microarray gene expression analysis

Gene expression was analyzed using 12 GeneChip (R) Mouse Gene 2.0 ST arrays representing 26,515 genes as previously described [35]. To minimize the differences of individual variability and increase the statistical power for the identification of potential biomarkers, microarray analysis was performed using equal amounts of purified RNA pooled from all of the study subjects (*N* = 18 per treatment group), and applied to 3 identical arrays from the same batch. Targets were prepared from pancreatic and adrenal tissues and microarrays were processed as described in the Affymetrix GeneChip Whole Transcript Expression Analysis manual using the Ambion WT expression kit and Affymetrix WT Terminal Labeling Kit as per manufacturers’ instructions. Briefly, approximately 100 ng adrenal and 500 ng pancreatic of total RNA was used to synthesize double-stranded DNA with random hexamers tagged with a T7 promoter sequence. Arrays were scanned using the Affymetrix 3000 7G scanner and GeneChip Operating Software version 1.4 to produce. CEL intensity files. This software also provided summary reports by which array QA metrics were evaluated including average background, average signal, and 3′/5′ expression ratios for spike-in controls, β-actin, and GAPDH. Microarray data was deposited at the MIAME compliant NCBI gene expression hybridization array data repository (GEO: http://ncbi.nlm.nih.gov/geo) under accession # GSE141313 and GSE141310 (expression data from pancreatic and adrenal tissue respectively).

### Quantitative PCR (qPCR) validation of microarray analysis

qRT-PCR was performed on a LightCycler 480 instrument (Roche Molecular Biochemicals, Mannheim, Germany) using the Hot start reaction mix for SYBR Green I master mix, (Roche) as previously described [37]. Amplifications were according to cycling conditions suggested for the LightCycler 480 instrument in the SYBR Green Master Mix handbook (initial activation at 95 °C for 5 min; 45 cycles of 94 °C for 15 s, primer dependent annealing temperature for 20 s, 72 °C for 20 s). All PCR reactions were performed in triplicate using cDNA synthesized from the same batch and starting amount of total RNA. Primer pairs were synthesized in a local facility in our institution and used at a final concentration of 1 μM (microM). A complete list of the genes and primer sequences are detailed in Supplemental Table s1. Relative gene expression values were analyzed using the 2^−ΔΔCT method [38]. Pearson correlation analysis between qPCR and microarray data were displayed using a scatter plot.

### Data analysis

Statistical analyses were performed using IBM SPSS statistics software version 20 (SPSS Inc., Chicago, IL) as previously described [27, 35]. Data were presented as means ± SEM for body characteristics and Insulin Tolerance test (ITT). Differential pancreatic and adrenal gene expression analysis were performed using the Partek Genomic suite software version 6.6 (Partek Incorporated, USA) using samples of either pancreatic or adrenal tissue pooled from mice (*N*=18, applied in triplicate) grouped by strain (KK/HlJ or C57BL/6 J) and sex (male or female). The probe set data were categorized and grouped by means of Principal Component Analysis (PCA) and Robust Multi-Array Average (RMA) algorithm was used for background correction [39] as implemented in the microarray analysis software (MAS). The standard RMA algorithm used the log 2 transformed perfect match (PM) values followed by quantile normalization. The transformed PM values were then summarized by median polish method. Probesets without unique Entrez gene identifiers were removed from further analysis and values below log 4 were filtered out. For identification of strain- and sex-dependent differentially expressed genes (DEGs) we used a 2-factor design (male KK/HlJ versus male C57BL/6 J; male KK/KlJ versus female KK/KlJ; female KK/KlJ versus female C57BL/6 J; male C57BL/6 J versus female C57BL/6 J) with significance set at *p*< 0.05. Regulated genes were identified using False Discovery Rate (FDR) method [40] in which *p*-values were adjusted simultaneously across multiple subgroup comparisons. The significant and differentially expressed genes were selected by means of cut-off fold change (>±1.4) and FDR-adjusted ANOVA *p-*value.

We next selected subsets of DEGs for further analysis which were expressed either in a strain-specific manner irrespective of sex, or sex-dependent irrespective of strain, using a fold-change cut-off of (>±1.4). Ingenuity Pathway Analysis (IPA) software (Ingenuity Systems, Redwood City, CA) was used to further analyze the functionality of the identified subsets. Genes with known gene symbols according to the Human Gene organization (HUGO) and their corresponding expression values were uploaded into the IPA software, where gene symbols were mapped to their corresponding gene object in the Ingenuity Pathways Knowledge Base (IPKB). To perform functional enrichment tests of the candidate genes, we used IPA for Gene Ontology (GO) term analysis of Biological Function and Diseases pathways, and the Database for Annotation, Visualization and Integration Discovery (DAVID) for Subcellular Compartment localization. Networks of potentially interacting DEGs were identified using IPA and placed into node-edge diagrams comprised of focus molecules (DEGs identified by the microarray analysis) and other interacting molecules. All the edges were supported by at least 1 reference from the scientific literature or from canonical information contained in the IPKB. Nodes were displayed using various shapes that represent the gene product evaluated by the scores which were generated through calculation in the IPA software and represented the significance of the molecules in the network. The analysis also included pathways with intermediate regulators that involve more than one link to create a comprehensive picture of the possible gene interactions.

## Results

### Glucose and insulin homeostasis

In addition to significantly heavier body weight together with greater pancreas and visceral adipose tissue weights, impaired glucose homeostasis was apparent in KK/HlJ mice of both sexes as demonstrated by a greater incremental change in the AUC _GLUCOSE_ during a random-fed Insulin Tolerance test administered at 18 weeks of age (*p* < 0.001,Table 1). Conversely, female C57BL/6 J mice had a higher first-order K^ITT^, shorter glucose T ^½^ and lower fasting serum glucose concentrations, indicative of a greater insulin sensitivity in these mice (*p* < 0.05). Both female and male KK/HlJ groups had overt hyperinsulinemia, with between 3.8- and 4.8-fold increases in serum insulin and C-peptide levels compared to the C57BL/6 J strain, accompanied by markedly elevated HOMA-IR levels.

**Table 1:** Strain -and-sex dependent differences in body variables and glucose homeostasis

	KK-M	KK-F	C57-M	C57-F	***p***-value strain^*^sex
Body weight (g)	37.11^**^ ± 0.58	36.99^**^ ± 1.04	28.79^**$$**^ ± 0.35	21.34 ±0.21	**< 0.001**
Visceral fat weight (g)	1.20^**^ ± 0.03	3.78^$$**^ ± 0.23	0.63^**$$**^ ± 0.04	0.27 ± 0.02	**< 0.001**
Pancreas weight (g)	0.33^*^ ±0.01	0.35^*^ ± 0.05	0.30^**$$**^ ± 0.01	0.22 ± 0.01	0.100
**Insulin Tolerance Test (ITT)**					
Basal Glucose (mg/dL)	251.33^$$$**^ ± 18.93	165.77^**^ ± 5.50	136.14 ± 4.28	130.83 ± 3.52	**< 0.001**
AUC_GLUCOSE_ (mg/dL.min)	13,145.2^$$$**^ ± 703.18	8790.41^**^ ± 371.24	7404.58^**$$**^ ± 214.05	6346.80 ± 299.54	**< 0.001**
K_ITT_ (%/min)	1.01 ± 0.11	1.07 ± 0.16	0.84 ± 0.07	1.22^**$$**^ ± 0.13	0.099
T1/2_GLUCOSE_ (min)	69.21 ± 8.24	69.65 ± 7.89	68.18^**$**^ ± 3.32	57.51 ± 6.31	0.720
**Fasting Blood measurements**					
Serum Glucose (mg/dL)	144.61 ± 5.28	144.42^**^ ± 4.61	138.86^**$$$**^ ± 3.67	119.67 ± 2.52	0.024
Serum Insulin (uIU/mL)	46.92^**^ ± 4.71	32.16^**^ ± 5.55	9.72 ± 0.54	8.51 ± 0.77	0.061
HOMA-IR	16.30^**^ ± 1.44	11.31^**^ ± 1.90	3.32^**$**^ ± 0.19	2.50 ± 0.23	0.076
C-Peptide	2134.30^$$$**^ ± 281.96	975.47^*^ ± 119.45	822.35^**$**^ ± 91.05	543.42 ± 96.07	**0.007**
Glucagon (pg/ml)	29.02 ± 4.43	157.65^*$$$^ ± 16.63	35.15 ± 4.66	96.93^**$$$**^ ± 9.73	**< 0.001**
Leptin	6360.02^**^ ± 305.11	6070.94^**^ ± 454.04	2439.69^**$$**^ ± 248.33	1291.04 ± 221.96	0.184
Corticosterone (ng/ml)	103.36 ± 10.40	200.74^**$$$**^ ± 21.42	92.32 ± 9.56	160.67^**$$**^ ± 17.86	0.351
Aldosterone (pg/ml)	649.63^***^ ± 50.60	571.13^***^ ± 45.26	329.75 ± 33.96	348.54 ± 31.15	**< 0.0001**
IL-6 (pg/ml)	62.02 ± 13.20	82.44^*^ ± 16.22	39.72 ± 4.64	31.08 ± 5.43	0.179
TNF-α (pg/ml)	8.07^*^ ± 0.96	9.25 ± 0.81	13.83^**$**^ ± 1.11	9.25 ± 1.56	**0.015**

Data presented as means ± SEM, *n*=18 per group

*p-values* for strain^*^sex were calculated using 2-way ANOVA

A significance of *p*-value < 0.05, < 0.001 and < 0.0001 based on independent t-tests is indicated by ^*^, ^**^ and ^***^ for significance between strains (C57-M vs KK-M and C57-F vs KK-F); and significant sex effect within each strain (i.e.C57-M vs C57-F and KK-M vs KK-F) is represented using ^**$**^, ^**$$**^ and ^**$$$**^ for *p* < 0.05, < 0.01 and < 0.001 respectively

*AUC*_***GLUCOSE***_ Area Under the Curve for glucose**,** *K*_*ITT*_ Rate of glucose utilization, T1/2 Half-life of glucose levels, *C57-M* C57BL/6 J Males, *C57-F* C57BL/6 J Females, *KK-M* KK/HIJ Males, *KK-F* KK/HIJ Females

Parts of data in Table 1 (rows 1–12, 16, 17) have been adapted from our related publication Inglis et al. (2019) [27]

### Selected serum hormone and pro-inflammatory cytokine analysis

Levels of glucagon and corticosterone were elevated in female mice of both strains; whereas serum leptin and aldosterone were elevated in KK/HlJ mice of both sexes (Table 1, *P*≤ 0.0001). Proinflammatory serum IL-6 levels were increased in the KK/HlJ strain, significantly (*P*≤0.05) in females and with a trend towards significance in males; whereas TNFα was mildly elevated only in male C57BL/6 J mice (Table 1, *P*= 0.015).

### Pancreatic gene expression

**Fig. 1:**
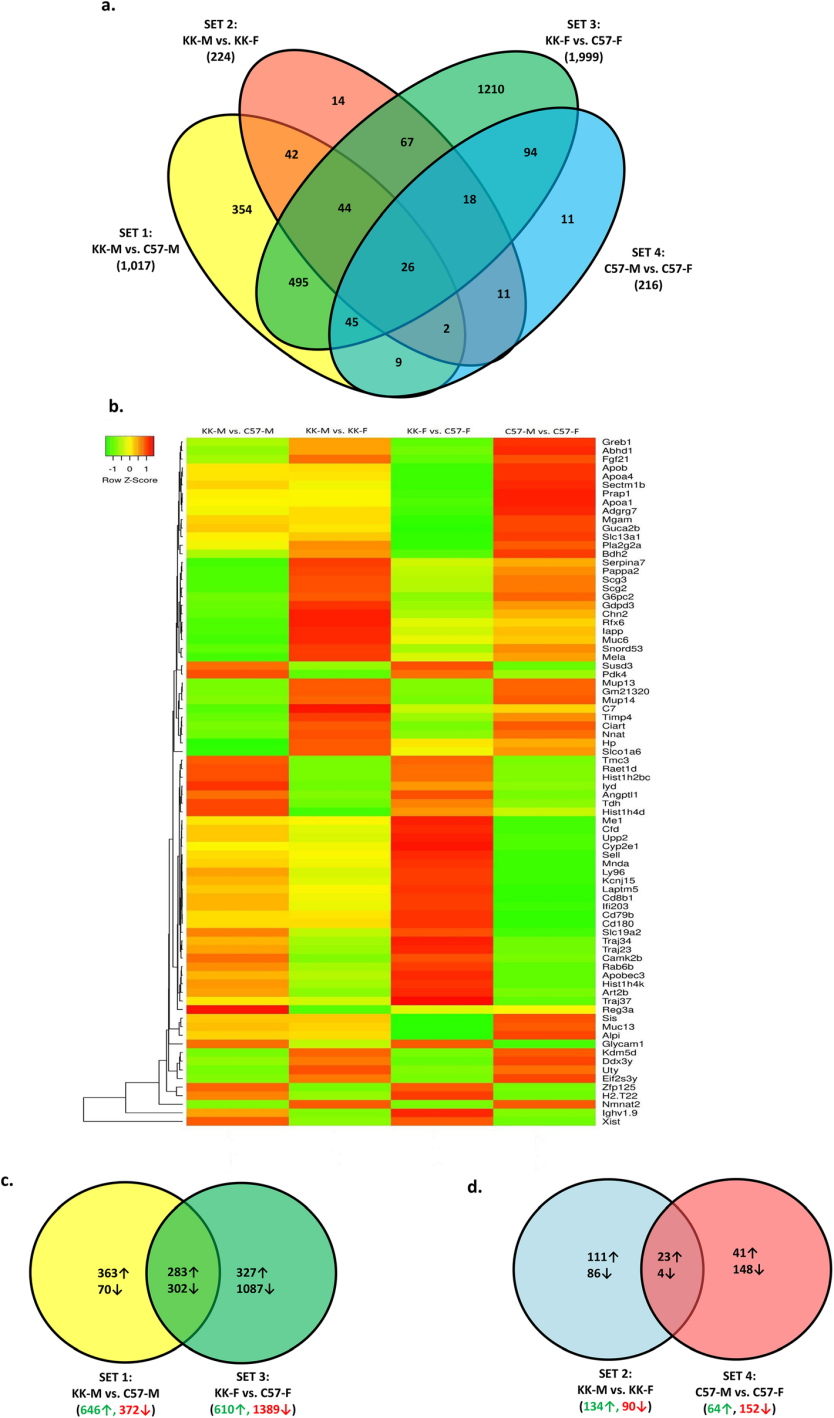
**a** 4-way Venn diagram analysis shows numbers of significant pancreatic differentially expressed genes (DEGs) and their overlapping categorization using multiple comparisons with fold-changes (FC≥± 1.4, FDR < 0.05) for Set 1: male KK/HlJ versus male C57BL/6 J (KK-M vs. C57-M, yellow); Set 2: male KK/HlJ versus female KK/HlJ (KK-M vs. KK-F, red), Set 3: female KK/HlJ versus female C57BL/6 J (KK-F vs. C57-F, green), and Set 4: male C57BL/6 J versus female C57BL/6 J (C57-M vs. C57-F, blue). **b** Heatmap with hierarchical clustering of differentially expressed pancreatic genes (FDR < 0.05) between the 4 Sets. Z-scores denote the relative gene expression levels with red and green representing high and low expression, respectively. **c** 2-way Venn diagram analysis of numbers of strain-biased pancreatic DEGs identified from the comparisons of Set 1 (KK-M vs. C57-M: yellow) and Set 3 (KK-F vs. C57-F: green), (FC≥± 1.4, FDR < 0.05). Green arrows represent up-regulated in KK-HlJ compared to C57BL/6 J, and red arrows represent down-regulated. **d** 2-way Venn diagram analysis of numbers of sex-biased pancreatic DEGs identified from the comparisons of Set 2 (KK-M vs. KK-F: blue) and Set 4 (C57-M vs. C57-F: red), (FC≥± 1.4, FDR < 0.05)

Affymetrix microarray analysis of sex- and strain-regulated differences in pancreatic gene expression was used in order to gain insight into the mechanisms underlying the observed variances in glucose and insulin homeostasis, as well as hormonal differences. We used a False Discovery Rate and a significance level set at 0.05 to identify 41,345 probesets for further analysis between the 4 experimental groups i.e., male (M) or female (F), KK/HlJ (KK) or C57BL/6 J (C57). In order to identify strain- and/or sex-specific subgroups, 2-factor ANOVA was applied to the four relevant contrast groups KK-M v C57M; KK-M v KK-F; KK-F v C57-F; C57M v C57-F, resulting in the detection of 10,269 gene with known identities. Genes were considered significant by applying a combined criterion for true expression differences consisting of ±> 1.4 fold change within a given contrast and a *P*-value < 0.05, and the numbers of DEGs between these contrast groups were depicted in a 4-way Venn diagram indicating the overlapping associations between significant DEGs of comparison groups (Fig. 1). In total, 1017 genes with significant expression differences between males of the KK/HlJ strain compared to the C57BL/6 J strain were identified in Set 1 (KK-M v C57M), whereas nearly twice as many genes (*N*=1999) exhibited strain-regulated expression in female mice (Set 3: KK-F v C57-F). Of the genes in Set 1, we found 495 genes which overlapped with genes exhibiting female-biased gene expression (Set 3), whereas the expression pattern of 354 genes were not common. Our analysis also showed fewer numbers of sex-biased pancreatic gene expression, with 224 KK/HlJ genes exhibiting sex-biased expression (Set 2: KK-M v KK-F) compared to 216 genes with similar expression patterns in C57BL/6 J mice (Set 4: C57M v C57-F). In the overlapping sets, there were only 26 significant genes detected between the 4 comparison groups. Overall, Set 3 (KK-F v C57-F) contained the highest number of pancreatic DEGs. In order to further illustrate the differences in pancreatic gene expression between the 4 groups, we used Partek hierarchical cluster analysis of the log transformed data to generate a heatmap depicting color-coded differences in the expression of the top 80 genes based on row z-scores using Euclidean distance measurements and average linkage method for clustering (Fig. 1b). In addition, pancreatic gene expression was further classified by differences in strain-biased expression (Fig. 1c) and sex-biased expression (Fig. 1d) for subsequent analysis.

#### Strain-related pancreatic gene expression

**Fig. 2:**
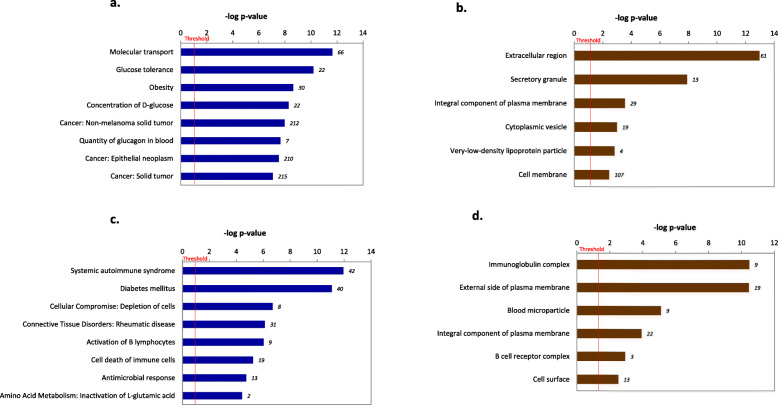
Gene Ontology Enrichment analysis of strain-associated pancreatic DEGs between the contrast groups (Set 1: KK-M vs. C57-M) and (Set 3: KK-F vs. C57-F) DEGs ≥± 1.4-fold expression differences (P≤0.05) ranked according to (**a**) Biological function and Disease category and (**b**) Cellular Compartments analysis of DEGs upregulated in KK/HlJ mice and (**c**) Biological function and Disease (**d)** Cellular Compartments analysis of DEGs upregulated in C57BL/6 J mice. Significance level is scored as –log(*p-*value) from Fischers exact test

Differences in pancreatic gene expression could provide molecular insights into strain-related differences in glucocentric phenotypes. Figure 1c indicates that out of the 1017 genes exhibiting strain differences in male mice (Set 1), the majority (*N*=646) were up-regulated in the KK/HlJ strain, whereas in female mice (Set 3), more than twice as many genes (*N*=1389) were expressed at significantly higher levels in the C57BL/6 J strain. In order to understand more about the function of these strain-biased genes, we used Gene Ontology (GO)-enrichment analysis in the Biological Processes and Diseases category using the Ingenuity Pathways Knowledge Base (IPKB), and Cellular Compartment localization analysis using DAVID. Figure 2a indicates the top 8 biological functions and diseases associated with the strain-dependent DEGs which were increased in KK/HlJ relative to C57BL/6 J plotted as enrichment scores (−log [*p*-value]). In keeping with the differences in glucocentric physiology and serum analysis that were observed between the two strains (Table 1), top biological functions and disease ontologies associated with these pancreatic DEGs included molecular transport, glucose tolerance, obesity and cancer. Positionally, these genes mapped to cellular compartments including secretory, integral components of the plasma membrane and cytoplasmic vesicles. (Fig. 2b). Conversely, the top biological functions and diseases associated with pancreatic genes upregulated in the C57BL/6 J strain included systemic autoimmune syndrome, Diabetes and cellular compromise: degradation of cells (Fig. 2c). Cellular compartment analysis of DEGs upregulated in C57BL/6 J mice were mapped to immunoglobulin complex formation, plasma membrane and blood microparticles (Fig. 2d, *P*≤0.05).

**Fig. 3:**
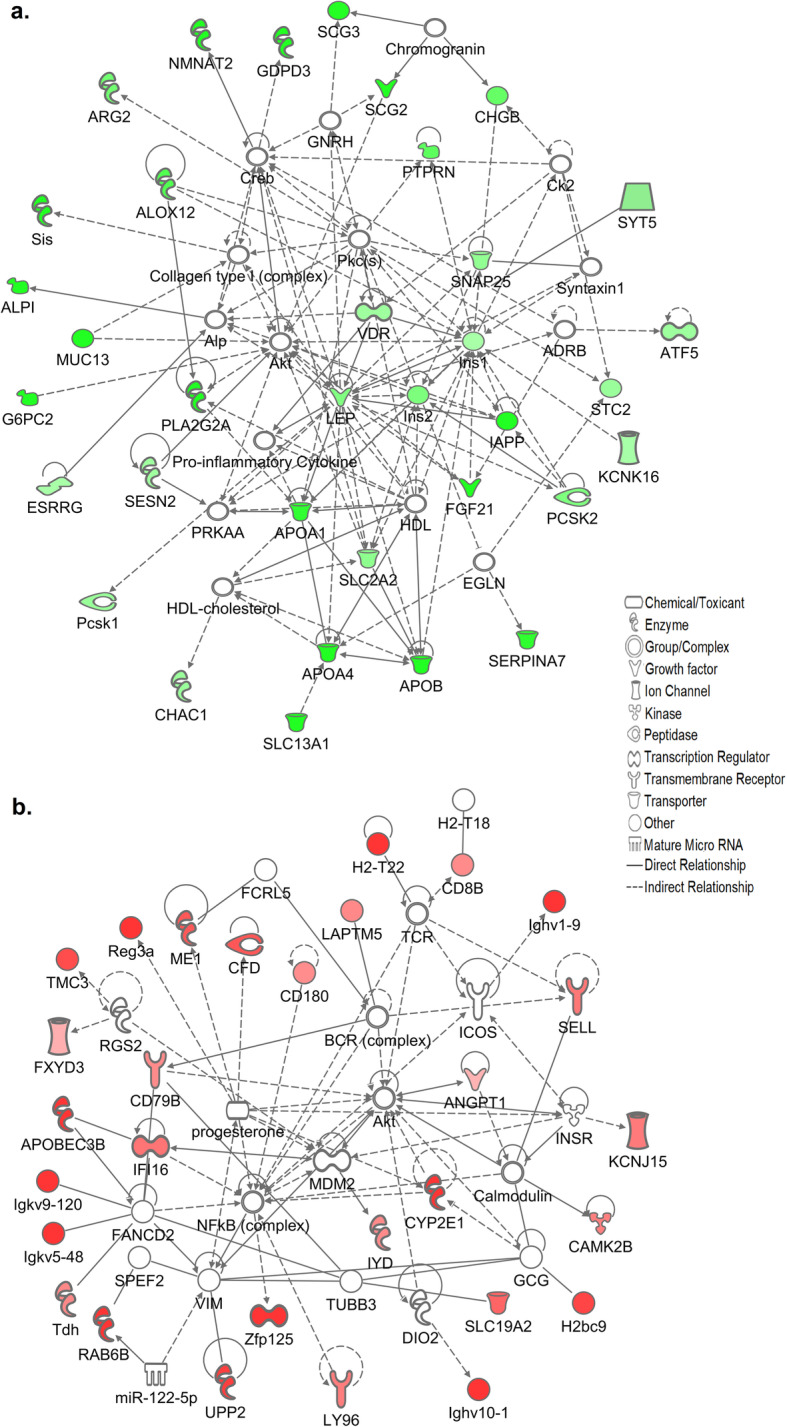
(**a**) Ingenuity pathway analysis (IPA) analysis of functional associations between the top scoring adrenal genes upregulated in the KK/HlJ strain according to magnitude of fold changes between the contrast groups: Set 1 (KK-M vs. C57-M) and Set 3 (KK-F vs. C57-F) ≥± 1.4-fold (*P*≤0.05). The shapes represent the molecular classes of the proteins and the intensity of the green-colored nodes represent the extent of upregulated expression, with dark green representing higher fold changes. Direct and indirect interactions are indicated by solid and dashed lines, respectively. (**b**) Top pancreatic network of genes upregulated in the C57BL/6 J strain, in which the intensity of the red-colored nodes represent the extent of upregulated expression

We next used IPA software to create a molecular network of functionally related pancreatic genes which were increased in the KK/HlJ strain based on highest fold changes (P≤0.05). Figure 3A indicates the top network of DEGs which were significantly upregulated in KK/HlJ mice of both sexes compared to C57BL/6 J by ≥± 1.4-fold. In agreement with the ~ 4-fold elevation in serum insulin levels that we observed in this strain, significant pancreatic up-regulated genes included many genes coding for proteins associated with the formation of β-cell insulin granules, also known as dense core secretory vesicles (DCSVs), including islet amyloid polypeptide (IAPP: Amylin), which was increased with respect to C57BL/6 J mice by an average of 4.51-fold in males and females. IAPP was functionally linked with insulin 1 (Ins1) and insulin 2 (Ins2), increased by an average of 2.04 and 3.06-fold; and Ins1 was functionally associated with a major βcell transcription and proliferation regulatory molecule: islet-specific transmembrane protein tyrosine phosphatase, receptor type, N (PTPRN, also known as IA-2), up-regulated by an average of 3.8-fold compared to the C57BL/6 J strain. Ins1 and PTPRN were functionally linked to key DCSV exocytosis-regulatory tSNARE molecule SNAP25 (synaptosomal-associated protein), upregulated by an average of 2.5-fold; as well as being linked to increased vitamin D receptor gene expression (VDR: 2.25-fold). Other molecules on this network and known to be associated with the biogenesis, regulation and function of pancreatic β-cell insulin granules included Secretogranins SCG2 and SCG3, the Staninocalcin STC2, Chromogranin CHGB, key diabetes susceptibility gene Proprotein Convertase Subtilisin/kexin type 2 (PCSK2), the synaptotagamin SYT5 and the outward rectifying potassium channel KCNK16 (TALK1), all upregulated in the KK/HlJ strain by between 1.51 and 6.75-fold. The network of highly upregulated pancreatic genes also included the major endocrine regulatory molecule fibroblast growth factor 21 (FGF21), increased by an average of 4.67-fold and functionally linked to IAPP; Apolipoprotein A4 (APOA4) linked to Apolipoprotein B and to Apolipoprotein A1, pro-inflammatory arachidonate 12-lipoxygenase (ALOX12: 4.10-fold) functionally linked to pancreatic Phospholipase A2 group IIA (PLA2G2A: 8.20-fold) and further linked to arginase type II (ARG2: 3.36-fold), the mitochondrial form of the enzyme which is known to be induced by obesity [41]. Strongly upregulated genes in the periphery of the network included the intracellular Golgi-associated NAD-synthesizing enzyme NMNAT2 (nicotinamide nucleotide adenylyltransferase 2: increased by an average of 23.53-fold), sucrase isomaltase (Sis: 10.47-fold), Mucin 13 (Muc13: 10.14-fold), solute carrier family 13a1 (SLC13A1: 5.76-fold) and serine peptidase inhibitor class A (SERPINA7: 3.23-fold).

Conversely, the top network of functionally-related pancreatic genes with the highest expression in the C57BL/6 J strain included many immune-related genes such as immunoglobulin heavy variable (Ighv) genes Ighv1–9, Ighv10–1 and Igkv9–120 and several other Ighv transcripts all of which were elevated from between 17.81- to 55.33-fold compared to the KK/HlJ strain (Fig. 3b, *P*≤0.05). In addition, several other immunologically-related transcripts were elevated in the pancreatic tissues of C57BL/6 J mice, including antigen-presenting H2-T22 (histocompatibility 2, T region locus 22: average of 17.34-fold), together with genes encoding cell surface recognition molecules CD180, CD79B and CD8b1.

Other significant pancreatic genes with elevated expression in the C57BL/6 J strain included zinc finger protein 125 (Zfp125: average of 11.92-fold) which was functionally linked to the apolipoprotein B gene APOBEC3B; CFD (complement factor D, adipsin: 3.88-fold), RAS oncogene RAB6B, (8.13-fold), regenerating islet-derived 3 alpha (Reg3a: increased by 4.44-fold), angiogenic ANGPT1 (Angiopoietin-1: 2.35-fold) and the potassium channel family gene KCNJ15 also increased by an average of 3.26-fold in male and female C57BL/6 J mice.

#### Sex-biased pancreatic gene expression

As stated previously, Fig. 1d indicates the Venn diagram analysis of 440 sex-dependent pancreatic gene expression in the two strains. We found a total of 224 genes with sex-biased expression of ≥± 1.4-fold in the pancreatic tissues from KK/HlJ mice, and 216 sex-biased genes in C57BL/6 J mice. In the KK/HlJ strain, there were 134 genes upregulated in males compared to 90 down-regulated, whereas in the C57BL/6 J strain we detected only 64 upregulated and 152 down-regulated DEGs. In order to be included as truly sex-biased, we looked for genes with a significant (*P*≤0.05) fold change of ≥± 1.4 in the ANOVA comparison of expressed genes in males from both the KK/HlJ and the C57BL/6 J strain compared to females from both strains. Within this constraint there were 27 qualifying pancreatic genes which included the Y-chromosome linked Eif2s3y, Uty and DDX3Y genes, all upregulated in males from both strains by between 11 and 18-fold compared to females (Table 2). Other genes with male-biased expression included twelve of the Major Urinary Proteins (MUPs) which were overexpressed in males by between 2.69–8.01-fold compared to females; as well as neuronatin (Nnat), lysine-specific demethylase 5D (KDM5D), Complement Component 7 (C7) and haptoglobin (HP). Genes with female-biased expression in both strains were far fewer but included the well-characterized x-chromosome linked gene XIST (X-inactive specific transcript), the cell adhesion molecule Glycam1, PDK4 (pyruvate dehydrogenase kinase, isoenzyme 4) and SUSD3 (Sushi Domain Containing 3), all with increased expression in females by between 1.7- and 98.7-fold (Table 2, *P*≤0.05).

**Table 2:** Sex specific DEGs in pancreas

Gene symbol	Gene name	Accession number	Average fold change
**Upregulated in male mice compared to female**			
Eif2s3y	Eukaryotic Translation Initiation Factor 2, Subunit 3, Structural gene Y-linked	NM_012011	16.85
Uty	Ubiquitously Transcribed Tetratricopeptide Repeat Gene, Y Chromosome	NM_009484	13.46
DDX3Y	Dead (Asp-Glu-Ala-Asp) Box Polypeptide 3, Y-Linked	NM_012008	11.85
KDM5D	Lysine (K)-Specific Demethylase 5D	NM_011419	10.54
Mup20	Major Urinary Protein 20	NM_001012323	8.01
Mup14	Major Urinary Protein 14	NM_001199999	5.98
Mup15	Major Urinary Protein 15	NM_001200004	5.91
Mup8	Major Urinary Protein 8	NM_001134676	5.91
C7	Complement Component 7	NM_001243837	5.82
Mup13	Major Urinary Protein 13	NM_001134674	5.52
Mup7	Major Urinary Protein 7	NM_001134675	5.21
Mup18	Major Urinary Protein 18	NM_001199333	4.74
Mup1	Major Urinary Protein 1	NM_001163010	4.71
Mup19	Major Urinary Protein 19	NM_001135127	4.61
Mup12	Major Urinary Protein 12	NM_001199995	4.42
Mup2	Major Urinary Protein 2	NM_001045550	4.40
Mup16	Major Urinary Protein 16	NM_001199936	4.27
Nnat	Neuronatin	NM_001291128	2.69
HP	Haptoglobin	NM_017370	2.01
Igkv1–88	Immunoglobulin Kappa Chain Variable 1–88	OTTMUST00000132310	2.01
TIMP4	Tissue Inhibitor of Metalloproteinase 4	NM_080639	1.97
CIART	Circadian Associated Repressor of Transcription	NM_001033302	1.45
Gm21320	Unknown	XM_003688783	5.26
**Downregulated in male mice compared to female**			
XIST	Inactive X Specific Transcripts	NR_001463	−98.73
Glycam1	Glycosylation Dependent Cell Adhesion Molecule 1	NM_001289587	−11.54
PDK4	Pyruvate Dehydrogenase Kinase, Isoenzyme 4	NM_013743	−2.57
SUSD3	Sushi Domain Containing 3	NM_025491	−1.75

*DEG* Differentially Expressed Gene

### Adrenal gene expression

**Fig. 4:**
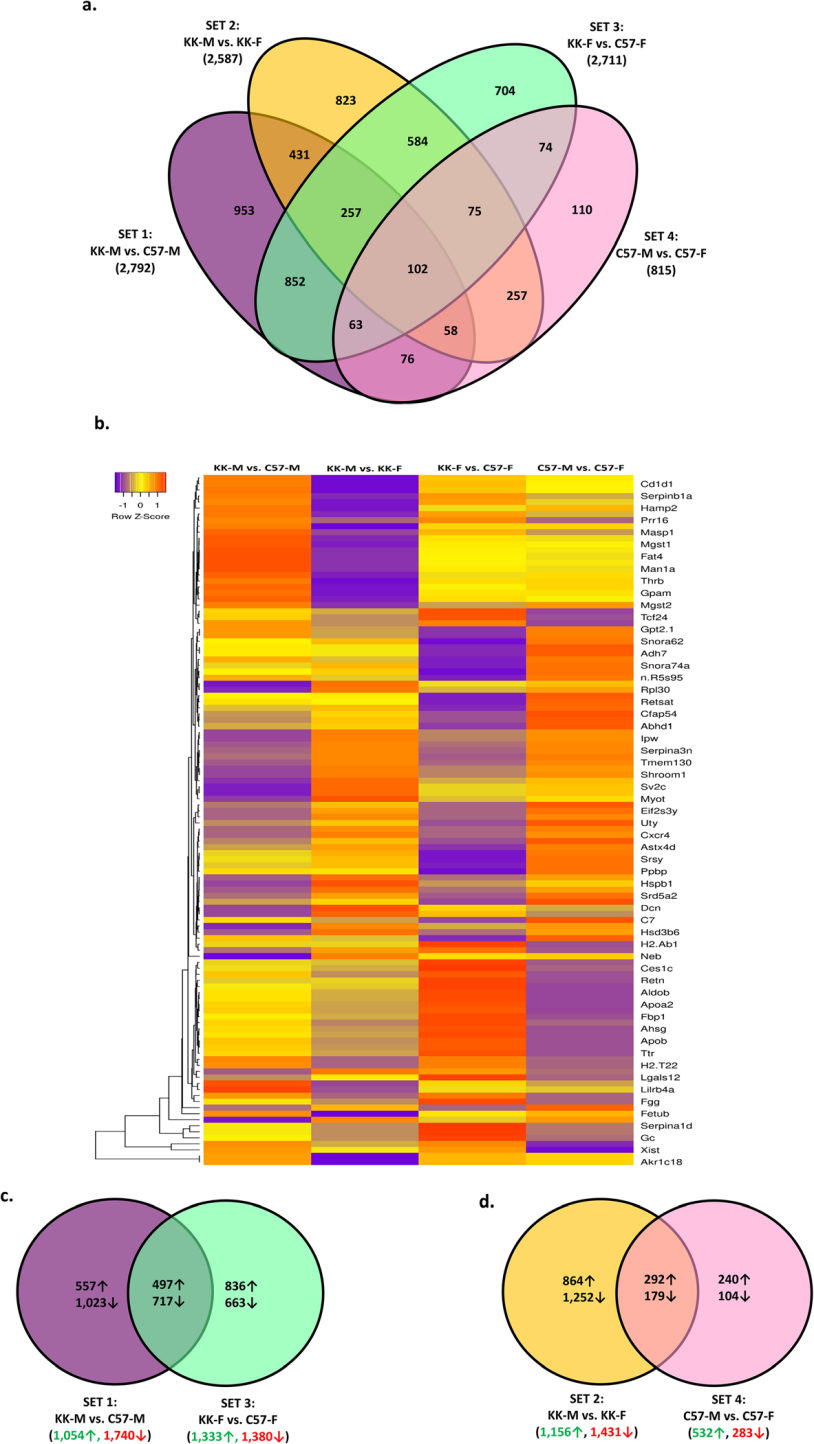
(**a**) 4-way Venn diagram analysis shows numbers of significant adrenal differentially expressed genes (DEGs) and their overlapping categorization using multiple comparisons with fold-changes (FC≥± 1.4, FDR < 0.05) for Set 1: male KK/HlJ versus male C57BL/6 J (KK-M vs. C57-M, violet); Set 2: male KK/HlJ versus female KK/HlJ (KK-M vs. KK-F, yellow), Set 3: female KK/HlJ versus female C57BL/6 J (KK-F vs. C57-F, green), and Set 4: male C57BL/6 J versus female C57BL/6 J (C57-M vs. C57-F, pink). (**b**) Heatmap with hierarchical clustering of differentially expressed adrenal genes (FDR < 0.05) between the 4 Sets. Z-scores denote the relative gene expression levels with red and violet representing high and low expression, respectively. (**c**) 2-way Venn diagram analysis of numbers of strain-biased adrenal DEGs identified from the comparisons of Set 1 (KK-M vs. C57-M: violet) and Set 3 (KK-F vs. C57-F: green), (FC≥± 1.4, FDR < 0.05). Green arrows represent up-regulated in KK-HlJ compared to C57Bl-6 J, and red arrows represent down-regulated. (**d**) 2-way Venn diagram analysis of numbers of sex-biased adrenal DEGs identified from the comparisons of Set 2 (KK-M vs. KK-F: yellow) and Set 4 (C57-M vs. C57-F: pink), (FC≥± 1.4, FDR < 0.05)

In addition to differences in adiposity and glucose homeostasis between the C57BL/6 J and KK/HlJ strain, our analysis indicated strain- and sex-dependent differences in serum corticosterone and aldosterone, both of which are adrenal-derived steroid hormones. For identification of strain- and sex-regulated adrenal genes we applied the same inclusion criteria i.e. expression differences consisting of ±> 1.4 fold change within a given contrast i.e. KK-M v C57M; KK-M v KK-F; KK-F v C57-F; C57M v C57-F, and a *P*-value of ≤0.05. This approach resulted in the detection of 10,338 DEGs which were used to generate a four-set Venn diagram in order to analyze the numbers of common and unique genes within this dataset (Fig. 4a, *P*≤0.05). The highest numbers of DEGs were found in Sets 1 (KK-M v C57-M: 2792 genes) and Set 3 (KK-F v C57-F: 2711 genes), indicating greater strain-related gene expression differences compared to sex-related differences in Sets 2 (KK-M v KK-F: 2587 genes) and Set 4 (C57-M vs C57-F: 815 genes). In the overlapping adrenal gene sets, there were 102 significant genes detected between the 4 comparison groups, and the highest number of genes were found in the subset of Set 1 and Set 3 (Fig. 4a, 852 genes, *P*≤0.05). Figure 4b shows a differentially expressed gene heatmap with z-score hierarchical clustering of genes based on DEGs with the greatest fold change difference in individual sets. The differences in adrenal gene expression between the four contrast groups were further classified using 2-way Venn diagrams of strain-biased (Fig. 4c) and sex-biased (Fig. 4d) expression for subsequent analysis.

#### Strain-related adrenal gene expression

**Fig. 5:**
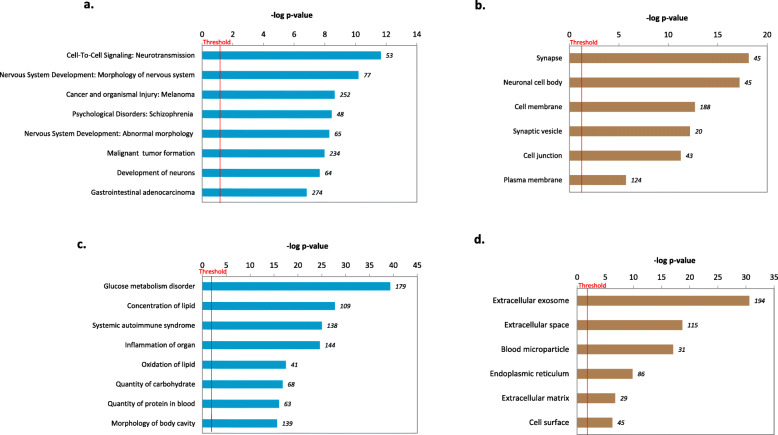
Gene Ontology Enrichment analysis of strain-associated adrenal DEGs between the contrast groups (Set 1: KK-M vs. C57-M) and (Set 3: KK-F vs. C57-F) DEGs ≥± 1.4-fold expression differences (P≤0.05) ranked according to (**a**) Biological function and Disease category and (**b**) Cellular Compartments analysis of DEGs upregulated in KK/HlJ mice and (**c**) Biological function and Disease category and (**d**) Cellular Compartments analysis of DEGs upregulated in C57BL/6 J mice. Significance level is scored as –log(*p*-value) from Fischers exact test

To better understand the unique gene expression patterns of male and female KK/HlJ and C57BL/6 J adrenal glands, and to look for any patterns of expression which could account for the adrenal hyperplasia and other changes reported by light microscopy studies [30], we next considered subgroups of strain-dependent DEGs, defined as being either up-regulated in both male and female KK/HlJ mice compared to male and female C57BL/6 J mice by a factor of ≥1.4-fold, or up-regulated in both male and female C57BL/6 J mice compared to male and female KK/HlJ mice. This resulted in a set of 497 adrenal genes upregulated in KK/HlJ mice compared to C57BL/6 J, and 717 which were down-regulated irrespective of sex (Fig. 4c), which were analyzed for enrichment of functional annotation using IPA. Figure 5a shows the top 8 Biological Functions and Diseases associated with these subsets, including cell-to-cell signaling: neurotransmission, nervous system development and cancer: melanoma categories. These genes compartmentalized primarily to the neuronal body, synapses and cell membranes, according to the DAVID database (Fig. 5b). Conversely, the top biological function and disease ontologies for adrenal genes upregulated in C57BL/6 J mice of both sexes included glucose metabolism disorders, concentration of lipid, and systemic autoimmune syndrome and inflammation (Fig. 5c). Top cellular compartments included the extracellular exome, extracellular space and blood microparticles (Fig. 5d, *P*≤0.05).

**Fig. 6:**
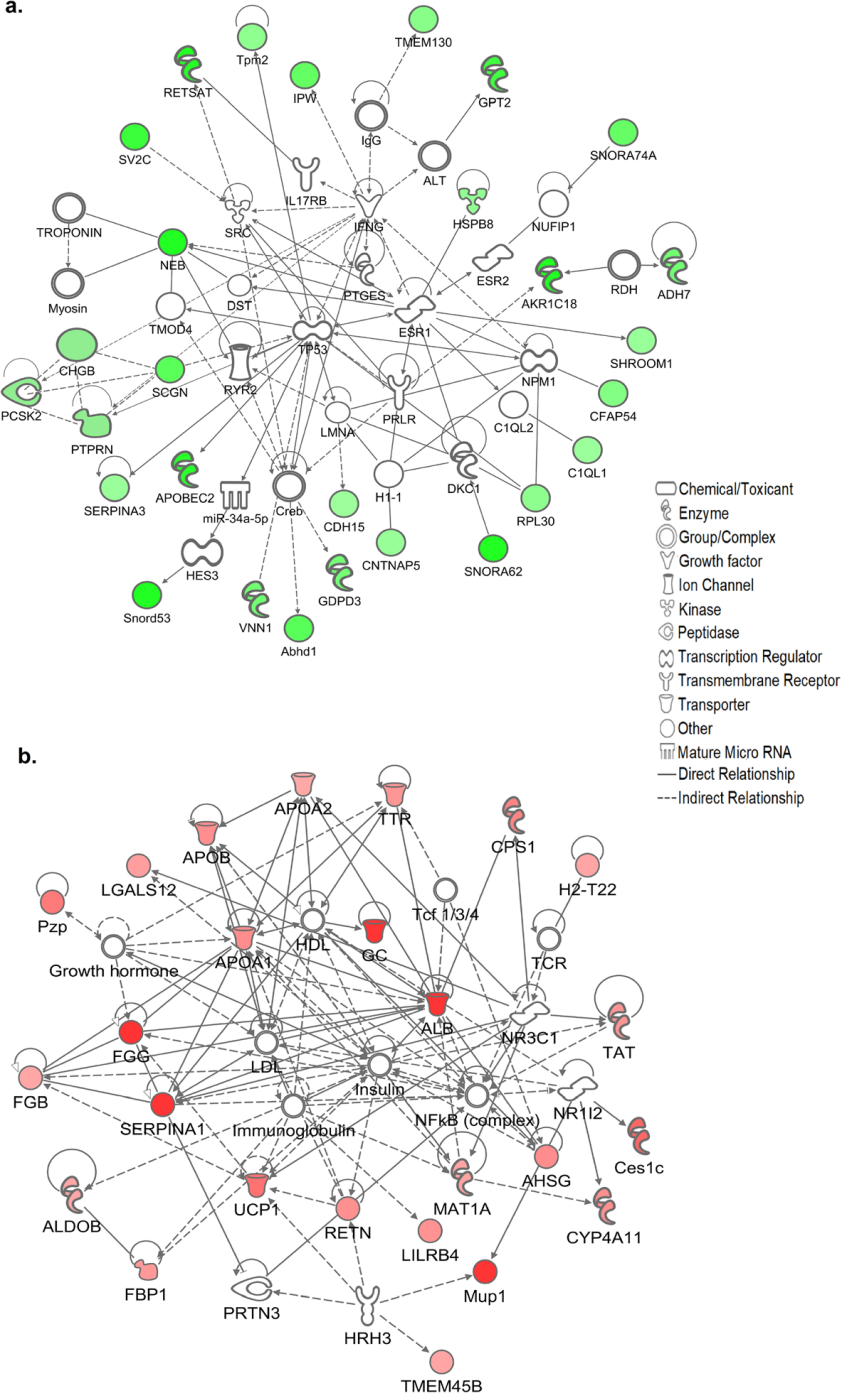
(**a**) Ingenuity pathway analysis (IPA) analysis of functional associations between the top scoring adrenal genes upregulated in the KK/HlJ strain according to magnitude of fold changes between the contrast groups: Set 1 (KK-M vs. C57-M) and Set 3 (KK-F vs. C57-F) ≥± 1.4-fold (*P*≤0.05). The shapes represent the molecular classes of the proteins and the intensity of the green-colored nodes represent the extent of upregulated expression, with dark green representing higher fold changes. Direct and indirect interactions are indicated by solid and dashed lines, respectively. (**b**) Top adrenal network of genes upregulated in the C57BL/6 J strain, in which the intensity of the red-colored nodes represent the extent of upregulated expression

To further identify key adrenal genes involved in this cellular function and establish the connectivity between these genes, we next used IPA software to create the top molecular networks of functionally related genes which were differentially expressed between the two strains based on highest fold changes. Figure 6a shows the top network of adrenal DEGs with significantly stronger expression in the KK/HlJ strain which includes the progesterone-catabolizing enzyme Akr1c18 (AKR1C3: 17β-HSD) which was elevated by 3.1-fold in male KK/HlJ and 15.8-fold in female KK/HlJ mice compared to the C57BL/6 J strain. We also found an increase in the metabolic marker oxidoreductase enzyme Retinol saturase (RETSAT), which was elevated by 2.3- and 7.4-fold in male and female KK/HlJ respectively. Other strain associated genes which were markedly increased in the KK/HlJ strain included the neuroendocrine marker secretagogin (SCGN: average of 5.38-fold increase), linked to the key dense core secretory vesicle (DCSV)-regulating chromogranin CHGB and to PTPRN; as well as the apolipoprotein B mRNA-editing enzyme APOBEC2 (average of 4.56-fold increase). Other noteworthy genes upregulated in the KK/HlJ strain included the membrane-anchored metabolic regulator vanin1 (VNN1) which has previously been shown to be involved in the development of adrenocortical neoplasia [42], the adrenocortical enzyme glutamic pyruvate transaminase 2 (GPT2), ribosomal protein RPL30, the glycerophosphodiester phosphodiesterase GDPD3 and several of the small nucleolar RNA, C/D box genes including Snord53, SNORA62, and SNORA74A.

Genes which were upregulated by greater than 5-fold in C57BL/6 J adrenal tissues of both sexes compared to the KK/HlJ strain included more than 10 of the Major Urinary Proteins such as Mup1, 2,3,8, 13,14 and 15; (Fig. 6b). We also found significant increases in the expression of the albumin gene (ALB) by between 21.5- and 80.7-fold, increased fibrinogen gamma chain (FGG) expression, as well as increased expression of several members of the serine peptidase inhibitor family: SERPINA1c, SERPINA1d and SERPINC1. Other noteworthy upregulated genes included Apolipoproteins A-1 (APOA1), A-II (APOA2) and Apolipoprotein B (APOB), Group specific component (GC: the gene encoding Vitamin D binding protein), carbamoylphosphate synthase 1 (CPS1), uncoupling protein 1 (UCP1) and proliferation marker TMEM45B.

#### Sex-related adrenal gene expression

Our analysis showed greater numbers of true sex-biased gene expression in the adrenal glands compared to the pancreatic tissue, with 471 genes exhibiting a significant (*P*≤0.05) fold change of ≥± 1.4 in the ANOVA comparison (Fig. 4d), which comprised of 292 genes with upreglated expression in the males of both strains compared to 179 gene with female-biased expression in both strains. Table 3 indicates the top 30 genes which were expressed at significantly higher levels in male adrenal tissues from both KK/HlJ and C57BL/6 J mice ranked by fold change, as well as the top 30 genes with female-biased expression in the 2 strains. We also found 14 transcripts with significant fold changes but without known gene names assigned. The highest ranking adrenal genes included several that were also overexpressed in a sex-biased pattern in the pancreatic tissue, such as Y-chromosome linked DDX3Y Eif2s3y and Uty, all upregulated in males from both strains by between 9 and 18-fold compared to females. Also greatly overexpressed in male adrenal tissues of both strains we found 4 genes encoding amplified spermatogenic transcripts X (Astx, Astx1c, Astx3 and Astx4d), male-specific histone demethylase lysine-specific demethylase 5D (KDM5D), LY6D, and the proteoglycan aggrecan (Acan: Tables 3, 8.52-fold, *P*≤0.05). Genes which are less-commonly associated with male-biased expression included matrix metallopeptidase-12 (MMP12), neuronatin (Nnat), Hydroxy-Delta-5-Steroid Dehydrogenase, 3 Beta gene (HSD3B6) and CXC motif chemokine receptor type 4 (CXCR4).

**Table 3:** Sex specific DEGs in adrenal

Gene symbol	Gene name	Accession number	Average fold change
**Upregulated in male mice compared to female**			
DDX3Y	Dead (Asp-Glu-Ala-Asp) Box Polypeptide 3, Y-Linked	NM_012008	18.36
Mup20	Major Urinary Protein 20	NM_001012323	12.92
Uty	Ubiquitously Transcribed Tetratricopeptide Repeat Gene, Y Chromosome	NM_009484	12.27
Eif2s3y	Eukaryotic Translation Initiation Factor 2, Subunit 3, Structural gene Y-linked	NM_012011	9.60
Acan	Aggrecan	NM_007424	8.52
KDM5D	Lysine (K)-Specific Demethylase 5D	NM_011419	8.24
LY6D	Lymphocyte Antigen 6 Complex, Locus D	NM_010742	5.42
Astx4d	Amplified Spermatogenic Transcripts X Encoded 4D	ENSMUST00000123136	5.17
CXCR4	Chemokine (C-X-C Motif) Receptor 4	NM_009911	4.96
Astx1c	Amplified Spermatogenic Transcripts X Encoded 1C	ENSMUST00000177724	4.92
HSD3B6	Hydroxy-Delta-5-Steroid Dehydrogenase, 3 Beta- And Steroid Delta Isomerase 6	NM_013821	4.87
APLN	Apelin	NM_013912	4.39
Astx3	Amplified Spermatogenic Transcripts X Encoded 3	ENSMUST00000123508	4.35
MYOT	Myotilin	NM_001033621	4.04
MMP12	Matrix metallopeptidase 12	NM_008605	3.75
Srsy	Serine-Rich, Secreted, Y-Linked	XR_106321	3.32
KCNK1	Potassium Channel, Subfamily K, Member 1	NM_008430	3.28
C7	Complement Component 7	NM_001243837	3.22
Astx	Amplified Spermatogenic Transcripts X Encoded	XR_878232	3.21
Ssty2	Spermiogenesis Specific Transcript on The Y2	NM_023546	3.20
SRD5A2	Steroid 5 Alpha-Reductase 2	NM_053188	3.17
ETNK2	Ethanolamine Kinase 2	NM_175443	2.93
HSPB1	Heat Shock Protein 1	NM_013560	2.90
Ppbp	Pro-Platelet Basic Protein	NM_023785	2.67
KCNK9	Potassium Channel, Subfamily K, Member 9	NM_001033876	2.64
SEMA5B	Sema Domain, Seven Thrombospondin Repeats (Type 1 And Type 1-Like)	NM_013661	2.62
VSNL1	Visinin-Like 1	NM_012038	2.62
FGF11	Fibroblast Growth Factor 11	NM_001291104	2.60
IGFBP5	Insulin-Like Growth Factor Binding Protein 5	NM_010518	2.57
Nnat	Neuronatin	NM_001291128	2.14
Gm29413	Unknown	ENSMUST00000186019	6.50
Gm21248	Unknown	XM_003689090	5.41
Gm20867	Unknown	NM_001160142	5.36
Gm20816	Unknown	NM_001160144	5.30
Gm20738	Unknown	NM_207162	5.12
Gm20823	Unknown	NM_001160143	5.03
Gm21242	Unknown	XM_003689089	4.75
**Downregulated in male mice compared to female**			
Akr1c18	Aldo-Keto Reductase Family 1, Member C18	NM_134066	−100.69
XIST	Inactive X Specific Transcripts	NR_001463	−93.61
AKR1D1	Aldo-Keto Reductase Family 1, Member D1	NM_145364	−70.31
FETUB	Fetuin Beta	NM_001083904	−14.88
CD1D1	CD1d1 Antigen	NM_007639	−10.79
C3	Complement Component 3	NM_009778	−9.92
SERPINB1	Serine (or Cysteine) Peptidase Inhibitor, Clade B, Member 1A	NM_025429	−8.71
Hamp2	Hepcidin Antimicrobial Peptide 2	NM_183257	−8.69
NREP	Neuronal Regeneration Related Protein	NM_001109988	−8.28
A2M	Alpha-2-Macroglobulin	NM_175628	−8.22
GPAM	Glycerol-3-Phosphate Acyltransferase, Mitochondrial	NM_008149	−6.20
Masp1	Mannan-Binding Lectin Serine Peptidase 1	NM_008555	−6.04
PRR16	Proline Rich 16	NM_001081224	−5.73
Vnn3	Vanin 3	NM_011979	−5.39
APPL2	Adaptor Protein, Phosphotyrosine Interaction, Ph Domain and Leucine	NM_145220	−5.14
FMO1	Flavin Containing Monooxygenase 1	NM_010231	−5.02
MGST2	Microsomal Glutathione S-Transferase 2	NM_001310482	−4.95
ELFN1	Leucine Rich Repeat and Fibronectin Type III, Extracellular 1	NM_175522	−4.88
ADH7	Alcohol Dehydrogenase 7 (Class IV), Mu or Sigma Polypeptide	NM_009626	−4.57
MAPK13	Mitogen-Activated Protein Kinase 13	NM_011950	−4.22
TCF24	Transcription Factor 24	NM_001285425	− 4.03
LRRK2	Leucine-Rich Repeat Kinase 2	NM_025730	− 4.02
MGST1	Microsomal Glutathione S-Transferase 1	NM_019946	−3.87
ST3GAL1	St3 Beta-Galactoside Alpha-2,3-Sialyltransferase 1	NM_009177	−3.85
CHKA	Choline Kinase Alpha	NM_001271496	−3.82
RFK	Riboflavin Kinase	NM_019437	−3.70
NR0B1	Nuclear Receptor Subfamily 0, Group B, Member 1	NM_007430	−3.66
THRB	Thyroid Hormone Receptor Beta	NM_001113417	−3.58
SPRY3	Sprouty Homolog 3 (Drosophila)	NM_001030293	−3.56
FAT4	Fat Tumor Suppressor Homolog 4 (Drosophila)	NM_183221	−3.47
Gm15883	Unknown	ENSMUST00000153562	−3.18
Gm24277	Unknown	ENSMUST00000158661	−3.08
Gm22866	Unknown	ENSMUST00000083947	−2.82
Gm20482	Unknown	XR_391144	−2.77
Gm19689	Unknown	NR_045094	−2.25
Gm15992	Unknown	ENSMUST00000130307	−2.25
Gm15895	Unknown	XR_388023	−2.19

*DEG* Differentially Expressed Gene

Table 3 also shows the top 30 genes with female-biased expression in adrenal tissue, based on fold-change magnitude. One of the genes with the highest level of female-biased expression was Akr1c18 (Aldo-Keto Reductase Family 1, Member C18, also known as 20α-hydroxysteroid dehydrogenase), in agreement with previous in situ hybridization studies [43]. Additionally we found a high female-bias in the expression of Xist, as well as Akr1D1 (Steroid 5β-reductase), FETUB (fetuin-B), Hamp2 (hepcidin antimicrobial peptide 2), NR0B1 (Nuclear Receptor Subfamily 0, Group B, Member 1, also known as Dax1) which is a key dominant-negative regulator of transcription; and GPAM (Glycerol-3-Phosphate Acyltransferase, mitochondrial).

#### Gene expression common to both tissues

**Fig. 7:**
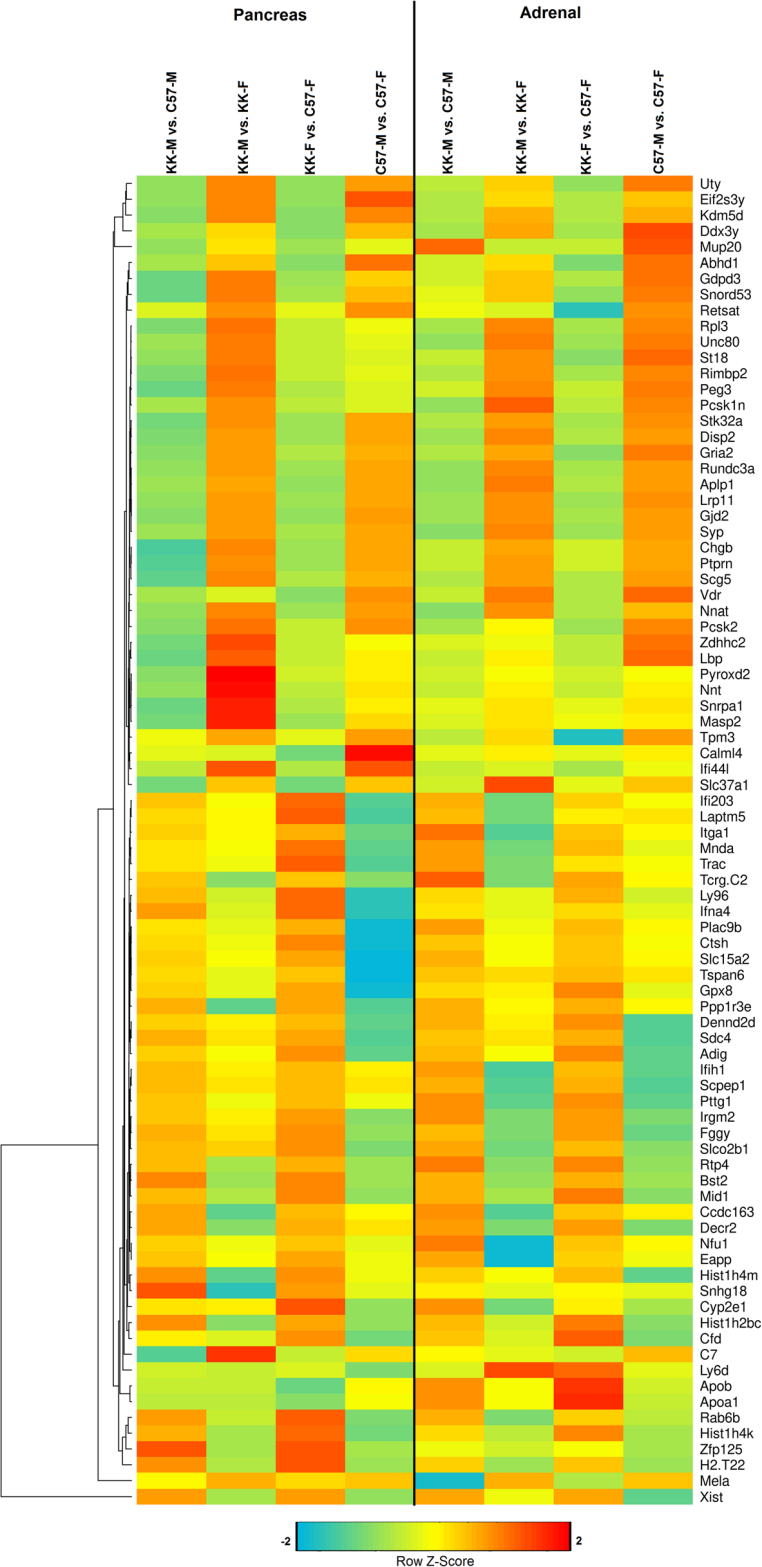
Heatmap with hierarchical clustering of differentially expressed genes common to both adrenal and pancreatic endocrine tissues which exhibited significant strain- or sex-biased expression (FDR < 0.05). Z-scores denote the relative gene expression levels with red and blue representing high and low expression, respectively

The final part of our microarray analysis was to examine the subset of genes which were differentially expressed between the two strains in both the adrenal and pancreatic tissues of male and female mice. Figure 7 shows a heatmap with z-score hierarchical clustering of genes with the highest differences in sex-biased or strain- associated expression in both the adrenal and pancreatic tissues identified by 2-factor ANOVA in the four comparisons KK-M v C57M; KK-M v KK-F; KK-F v C57-F; C57M v C57-F, and a *P*-value of ≤0.05. As expected the analysis showed a number of commonalities in sex-biased gene expression, with y-chromosomal genes Uty, DDX3Y, Eif2s3y, all exhibiting large differences in expression of between 10 and 22-fold in male adrenal and pancreatic tissues; whereas the only gene with significant female-biased expression common to both tissues was XIST, the gene encoding X inactivation-specific transcript. Other genes with male-biased expression of greater than 2-fold in both tissues and strains included neuroendocrine signaling molecule neuronatin (Nnat), the major urinary pheremone Mup20 (Darcin), and complement component 7 (C7) (Fig. 7, *P*≤0.05).

**Fig. 8:**
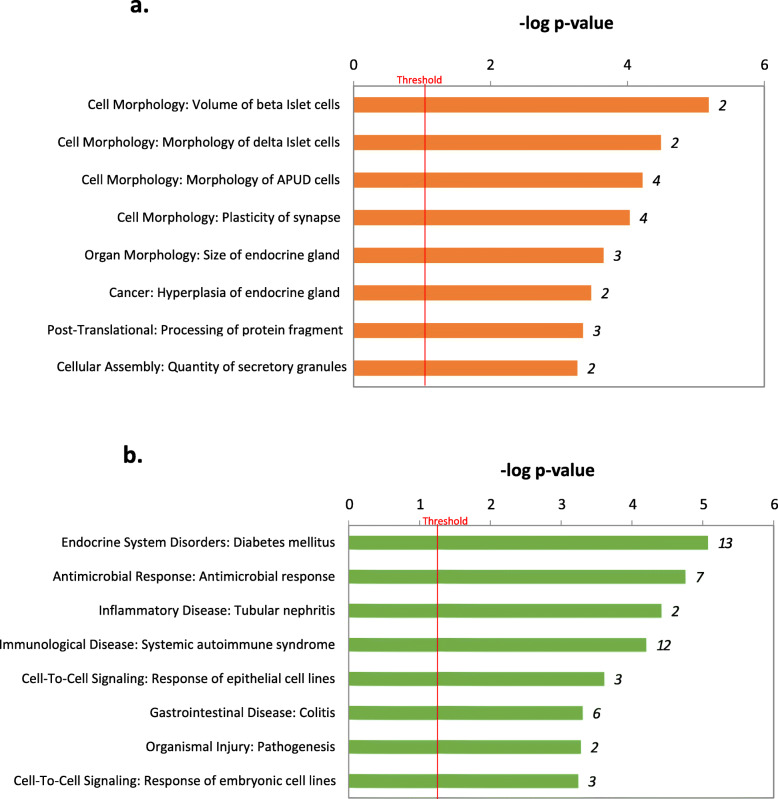
Gene Ontology Enrichment analysis of Biological function and Diseases associated with DEGs common to both pancreatic and adrenal tissues ranked according to significance. (**a**) Upregulated in the KK/HlJ strain; (**b**) Upregulated in the C57BL/6 J strain

Interestingly our analysis detected more than 60 genes with strain-biased expression common to both the pancreas and adrenal endocrine tissues, as shown in Table 4 in which only genes with an averaged fold change are classified as being either (a) significantly upregulated in both endocrine tissues of KK/HlJ mice by ≥1.5-fold (*N*=32), or (b) upregulated in both the pancreas and adrenal glands of C57BL/6 J mice by ≥1.5-fold (*N*=36). The gene ontologies of these common genes are indicated in Fig. 8a which shows the top Biological functions and Disease categories of pancreatic and adrenal genes which were commonly upregulated in KK/HlJ mice of both sexes, and included the categories of control of the volume and morphology of pancreatic Islet beta, delta and APUD (endocrine polypeptide) cells as well as development of synaptic plasticity. Conversely, the ontology of pancreatic and adrenal genes which were commonly upregulated in C57BL/6 J mice included genes involved in the development of Diabetes, the antimicrobial response and inflammatory conditions (Fig. 8b).

**Table 4:** Common strain specific DEGs in adrenal and pancreas

Gene symbol	Gene name	Accession number	Average fold change
**Upregulated genes in KK/HlJ mice compared to C57BL/6 J mice**			
Mela	Melanoma Antigen, Gal-Pol Polyprotein	BC113756	−32.57
Snord53	Small Nucleolar RNA, C/D Box 53	NR_028551	−8.01
GDPD3	Glycerophosphodiester Phosphodiesterase Domain Containing 3	NM_024228	−5.97
Abhd1	Abhydrolase Domain Containing 1	NM_021304	−5.00
RETSAT	Retinol Saturase	NM_026159	−4.85
SLC37A1	Solute Carrier Family 37 (Glycerol-3-Phosphate Transporter)	NM_001242427	−4.17
PTPRN	Protein Tyrosine Phosphatase, Receptor Type, N	NM_008985	−3.83
CHGB	Chromogranin B	NM_007694	−3.60
LBP	Lipopolysaccharide Binding Protein	NM_008489	−3.13
PCSK2	Proprotein Convertase Subtilisin/Kexin type 2	NM_008792	−3.12
MASP2	Mannan-binding Lectin Serine Peptidase 2	NM_001003893	−2.93
STK32A	Serine/Threonine Kinase 32A	NM_178749	−2.69
TPM3	Tropomyosin 3, gamma	BC092045	−2.67
Nnt	Nicotinamide Nucleotide Transhydrogenase	NM_001308506	−2.33
DISP2	Dispatched Homolog 2 (Drosophila)	NM_170593	−2.32
UNC80	Unc-80 Homolog	NM_175510	−2.31
RPL3	Ribosomal Protein L3	BC009655	−2.29
SCG5	Secretogranin V	NM_009162	−2.28
GRIA2	Glutamate Receptor, Ionotropic, AMPA2 (alpha 2)	NM_001039195	−2.06
RIMBP2	RIMS Binding Protein 2	NM_001081388	−2.00
ST18	Suppression of Tumorigenicity 18	NM_001244692	−1.89
PCSK1N	Proprotein Convertase Subtilisin/Kexin Type 1 Inhibitor	NM_013892	−1.80
APLP1	Amyloid Beta (A4) Precursor-Like Protein 1	NM_007467	−1.79
RUNDC3A	RUN Domain Containing 3A	NM_001252347	−1.64
LRP11	Low Density Lipoprotein Receptor-Related Protein 11	NM_172784	−1.55
BC023719	Unknown	BC023719	−27.64
BC018473	Unknown	BC018473	−15.31
LOC100861738	Unknown	LOC100861738	−4.22
1700020D05Rik	Unknown	1700020D05Rik	−3.67
Gm3264	Unknown	Gm3264	−2.85
Gm9855	Unknown	Gm9855	−2.79
Gm3383	Unknown	NM_001291093	−2.94
**Upregulated genes in C57BL/6 J mice compared to KK/HlJ mice**			
Igkv10–96	Immunoglobulin Kappa Variable 10–96	OTTMUST00000132012	136.82
H2-T22	Histocompatibility 2, T Region Locus 22	NM_010397	17.34
HIST1H4K	Histone Cluster 1, H4k	NM_178211	9.06
GABRA3	Gamma-Aminobutyric Acid (GABA) A Receptor, Subunit Alpha 3	NM_008067	8.32
RAB6B	RAB6B, Member RAS Oncogene Family	NM_173781	8.13
HIST1H2BC	Histone Cluster 1, H2bc	NM_023422	5.59
CFD	Adipsin Complement Factor D	NM_001291915	5.32
CYP2E1	Cytochrome P450, Family 2, Subfamily E, Polypeptide 1	NM_021282	4.34
LY96	Lymphocyte Antigen 96	NM_001159711	3.39
Trac	T-cell Receptor Alpha Constant	OTTMUST00000134701	3.37
Tcrg-C2	T-cell Receptor Gamma, Constant 2	OTTMUST00000134792	3.34
Mnda	Myeloid Cell Nuclear Differentiation Antigen	NM_001033450	3.26
Ifna4	Interferon Alpha 4	NM_010504	2.95
PTTG1	Pituitary Tumor-Transforming Gene 1	NM_001131054	2.88
IRGM2	Immunity-Related GTPase Family M Member 2	NM_019440	2.76
LAPTM5	Lysosomal-Associated Protein Transmembrane 5	NM_010686	2.73
HIST1H4M	Histone Cluster 1, H4m	NM_001195421	2.70
Adig	Adipogenin	NM_145635	2.65
ITGA1	Integrin Alpha 1	NM_001033228	2.62
MID1	Midline 1	NM_001290504	2.60
RTP4	Receptor Transporter Protein 4	NM_023386	2.43
NFU1	NFU1 Iron-Sulfur Cluster Scaffold Homolog	NM_001170591	2.14
GPX8	Glutathione Peroxidase 8 (Putative)	NM_027127	2.13
CTSH	Cathepsin H	NM_001312649	2.13
DECR2	2–4-Dienoyl-Coenzyme A Reductase 2, Peroxisomal	NM_011933	2.12
IFIH1	Interferon Induced with Helicase C Domain 1	NM_001164477	1.99
EAPP	E2F-Associated Phosphoprotein	NM_025456	1.85
DENND2D	DENN/MADD Domain Containing 2D	NM_001093754	1.79
TSPAN6	Tetraspanin 6	NM_019656	1.77
SCPEP1	Serine Carboxypeptidase 1	NM_029023	1.62
9930111J21Rik2	Unknown	9930111J21Rik2	10.41
Gm38463	Unknown	Gm38463	5.93
4930481A15Rik	Unknown	4930481A15Rik	4.29
2610507I01Rik	Unknown	2610507I01Rik	4.29
5830417I10Rik	Unknown	5830417I10Rik	4.25
Gm20236	Unknown	Gm20236	3.68

*DEG* Differentially Expressed Gene

**Fig. 9:**
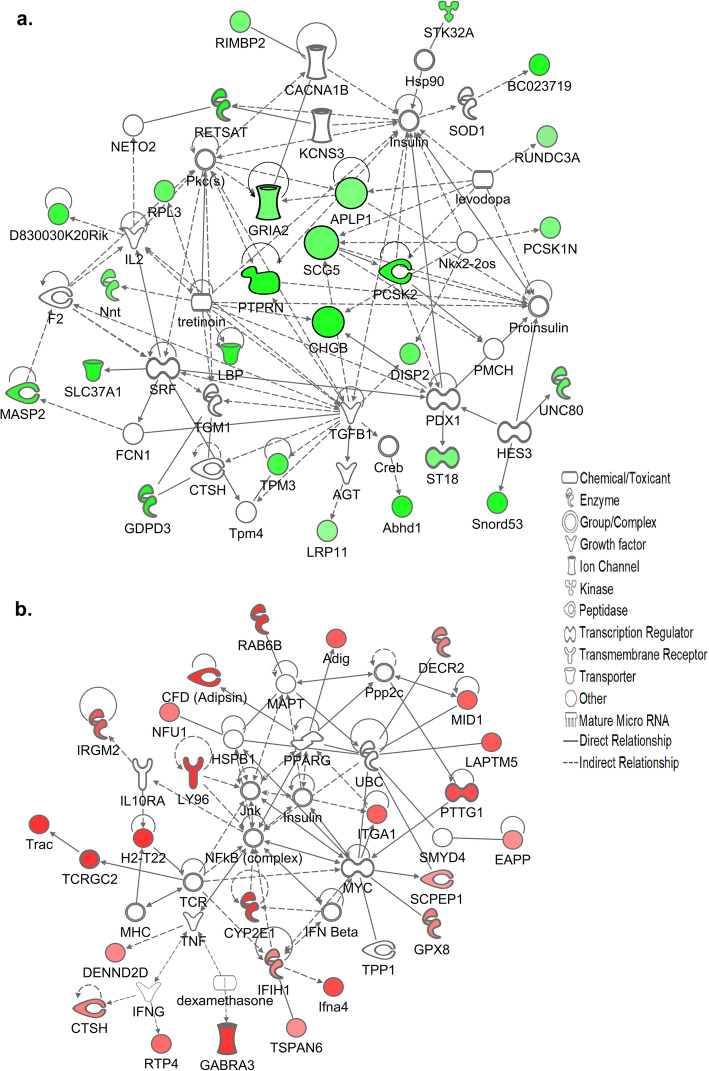
Functional network associations between the top scoring genes shared by both pancreatic and adrenal tissues (**a**) upregulated in the KK/HlJ strain; **b** upregulated in the C57BL/6 J strain, in which the intensity of the colored nodes represent the extent of upregulated expression

We were interested to establish the functional relationships between these commonly upregulated pancreatic and adrenal genes. Figure 9a indicates the top network generated from genes upregulated in the KK/HlJ strain in both tissues based on magnitude of fold change, and featured DCSV-associated PCSK2, increased by an average of 3.12-fold; PCSK1N, upregulated by 1.80-fold, together with chromogranin CHGB upregulated by 3.6-fold and SCG5, the gene encoding molecular chaperone secretogranin 5 upregulated by an average of 2.28-fold. Key DCSV membrane protein PTPRN (IA-2) was functionally linked to the glutamate ionotropic AMPA receptor GRIA2 (GluA2) via the neuronal cell adhesion gene APLP1, otherwise known as amyloid beta (A4) precursor-like protein 1. Our analysis also showed that although PTPRN was elevated in KK/HlJ mice by an average of 3.8-fold (Table 4), we did not detect a significant increase in PTPRN2 (IA-2β), a second autoantigenic member of the protein tyrosine phosphatase family encoded on a different chromosome (data not shown). Other networked genes with increased expression in KK/HlJ adrenal and pancreatic tissues of both males and females included retinol saturase (RETSAT), unc-80 homolog (UNC80), small nucleolar RNA, C/D box 53 (Snord53), lipopolysaccharide binding protein LBP, mannan-binding lectin serine peptidase 2 (MASP2) and DISP2 (dispatched homolog 2), all upregulated by an average of 2 to 8-fold.

Genes with higher expression in the pancreata and adrenal glands of C57BL/6 J mice of both sexes were ranked by fold change and the highest genes were functionally networked in Fig. 9b. This network included gamma-aminobutyric acid A receptor, subunit alpha 3 (GABRA3: 8.32-fold increase) linked to small GTPase RAB6B (8.13-fold increase); IFIH1 (Interferon induced with helicase C domain 1, also known as MDA5: 1.99-fold) linked to CFD (adipsin: 5.32-fold) and to Ifna4 (Interferon alpha 4:2.95-fold) and H2-T22 ((histocompatibility 2, T region locus 22:17.34-fold) and TSPAN6 (tetraspanin 6: 1.77-fold). Other notable genes upregulated in C57BL/6 J mice of both sexes and mapped to this network included the peroxisomal inflammatory marker DECR2 (2–4-dienoyl-Coenzyme A reductase 2: increased by 2.12-fold) functionally linked to Adig (Adipogenin: 2.65-fold); and H2BC4 (Histone Cluster 1 H2B Family Member C), which was functionally linked to TNFα. Our analysis also identified 13 strain-associated DEGs common to both tissues and sexes, with predicted gene identification numbers but without recognized gene names (listed in Table 4 for reference).

### Validation of microarray analysis using qRT-PCR

**Fig. 10:**
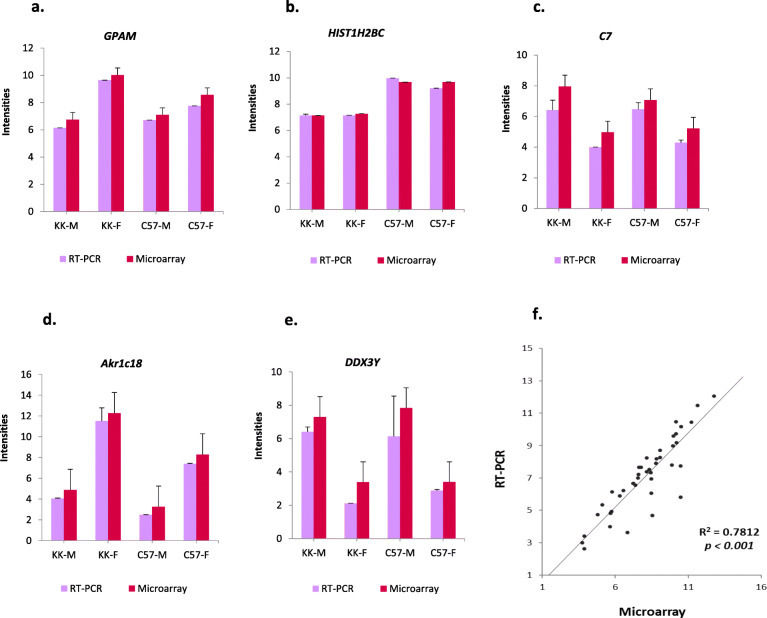
Expression plots of selected genes between qRT-PCR and Microarray. (**a**) GPAM: Glycerol-3-Phosphate Acyltransferase; (**b**) HIST1H2BC (Histone cluster 1, H2bc); (**c**) C7 (Complement factor 7); (**d**) Akr1C18 (Aldo-Keto Reductase Family 1, Member C18); (**e**) DDX3Y (DEAD-Box Helicase 3 Y-Linked). Significance between the four groups is represented as * at *P* value of ≤0.05. (**f**) Scatter-plot presentation of changes in expression of 16 selected genes as measured by qRT-PCR and microarray

In addition to our serum analysis which included insulin and related pancreatic and adrenal hormones, we used quantitative real-time PCR (qRT-PCR) in order to confirm our microarray results, using a selection of 25 pancreatic and adrenal genes randomly chosen based on biological relevance (Fig. 10a-f). A complete list of these genes and the Primer sequences are inventoried in Supplementary file S1. Pearson correlation coefficients between the microarray analysis and qRT-PCR were calculated and displayed as a scatter plot (Fig. 10f, *R*^2^=0.7812, *P*≤0.001).

## Discussion

Small-animal models of diabesity are an important and cost-effective tool in the scientific investigation of the global increase in obesity and diabetes. Our analysis of strain- and sex-based differences in pancreatic and adrenal gene expression is a continuation of our previous research on the physiological and behavioral differences between these 2 strains in terms of their usefulness as rodent models of the pathogenesis and treatment of these conditions. To our knowledge this is the first systematic analysis of gene expression differences, and the data complements previous light microscopic and morphometric studies concerning involvement of the pancreatic and adrenal glands in the etiology of diabesity [29, 30]. Our analysis confirms previous findings that KK/HlJ mice are hyperinsulinemic [29], and one of the most interesting findings was that the pancreata of KK/HlJ mice expressed higher levels of more than 10 well-characterized genes involved in the formation and function of β-cell insulin granules: heterogeneous populations of dynamic membrane-bound vesicles loaded with insulin, but also composed of over 150 proteins which act as a signaling node and metabolic sensor for the biogenesis, transport and storage of insulin [44]. Insulin granules store their cargo in at least two functionally distinct compartments: a fluid suspension containing many small molecules and proteins, and a dense core composed primarily of insulin and zinc. Early studies by Nakamura and Yamada described an increase in the number of pancreatic islets compared to the C57BL/6 J strain together with a 4-fold elevation in pancreatic insulin bioactivity [29]; and in addition to a similar elevation in serum insulin we found a 2 to 3-fold higher level of pancreatic insulin (Ins1 and Ins2) gene expression, as well increased levels of transcripts coding for secretory molecule Islet amyloid polypeptide (IAPP), a 37-residue hormone peptide which contributes towards glucose homeostasis by inhibiting glucagon release and regulate insulin secretion [45]. Other insulin granule-associated genes previously identified by proteomic studies [46] and upregulated in the KK/HlJ mice pancreata included key pro-IAPP processing enzyme PCSK2, together with the diabetes islet cell autoantigen Protein tyrosine phosphatase receptor type N (PTPRN, IA-2), Synaptosomal-Associated Protein SNAP25, Synaptotagamin SYT5, Chromogranin CHGB, Secretogranins SCG2 and SCG3, the Staninocalcin STC2, and the outward rectifying potassium channel KCNK16. We also noted a substantial increase in the expression of pancreatic NMNAT2 (Nicotinamide mononucleotide adenylyltransferase 2) in these mice. Although the exact role(s) of NMNAT2 have yet to be fully elucidated, there is considerable evidence that it may promote cancer cell survival by accelerating glycolysis via a mechanism that includes a reduction in the expression of the transcriptional regulator sirtuin SIRT6 [47]. This suggests a proliferative role for NMNAT2, and indeed previous studies have indicated that the pancreas of KK/HlJ mice exhibited hyperplasia [28, 29] although to date this is the first indication of the involvement of NMNAT2 in pancreatic hyperplasia. Taken together this would suggest that there is a good functional relationship between the observed strain-associated differences in pancreatic gene expression and the physiological differences associated with the KK/HlJ strain documented in Table 1 and detailed in other relevant publications [27–30].

Our analysis indicated an increase in serum IL-6 in addition to the observed increase in visceral fat in KK/HlJ mice. Previous studies have shown a direct correlation between epididymal adipose tissue and inflammatory status [48]; and pro-inflammatory markers present as highly expressed in our KK/HlJ pancreatic gene network included ALOX12, PLA2G2A, MUC13 and ARG2 all of which have been associated with inflammatory paradigms in previous research [49–51], and all of which have been shown to play various roles in pancreatic inflammation, oxidative stress and glucose metabolism [52–55].

We observed a very different microarray profile of up-regulated DEGs in the pancreata of C57BL/6 J mice. Amongst those genes with the highest fold change differences between the two strains, genes encoding immunoglobulin G heavy chain variable (Ighv) region were prominent, as well as genes of the immunoglobulin Kappa (κ) Locus. Studies by Tong & Liu [56] have shown that IgG-positive cells comprised about 1.4% of the total pancreatic cells in mice forming a thin septum surrounding the pancreatic ducts; and as with humans there are distinct differences in the repertoire of Ighv and Igκ variable sequences between inbred mouse strains [57]. Interestingly, differential expression of the adipokine complement factor D (CFD: adipsin) was elevated in C57BL/6 J mice, and other studies have shown that not only does CFD regulate the alternative complement pathway by generating complement component C3a, but it also augments pancreatic β-cell insulin secretion in vivo [58], suggesting a key role in glucose homeostasis.

We were interested to ascertain the identities of strain-biased genes common to both pancreatic and adrenal endocrine tissues, and whether we could identify any functional relationships between these DEGs. Amongst those upregulated in the KK/HlJ strain we identified a network of over a dozen functionally linked genes with 2-fold or higher increases in expression compared to the C57BL/6 J strain including the proprotein convertase subtilisin/kexin type 2 (PCSK2), found within dense core secretory vesicles (DCSVs) of neuroendocrine tissues including the adrenal and pancreatic glands, where it is known to be involved in the cleavage and activation of several hormones and neuropeptides [59]. We also found increases in the diabetes-associated Islet-cell autoantigen PTPRN, a 60-kDa type 1 membrane protein associated with the pancreatic and adrenal DCSVs [60, 61] together with Chromogranin B, a master regulator of DCSV biogenesis and function [62]. These three DEGs seemed to form a functional cluster common to both adrenal and pancreatic tissues in our microarray analysis and point to an increase in the number of DCSVs in the neuroendocrine tissues of KK/HlJ mice. In the case of the pancreas these would contain insulin and zinc, whereas in adrenal chromaffin cells the cargo would consist of catecholamines, neuropeptides and also micro RNAs [63]. Evidence for an increase in KK-mouse pancreatic and adrenal vesicular content is provided by earlier light microscopy studies [29, 30]. Functional deletion of PTPRN (IA-2) in mice results in impaired secretion of insulin, whereas overexpression leads to an increase in DCSVs and insulin secretion [64], which may have contributed to the hyperinsulinemia that we [27] and others [29] have observed in the KK/HlJ strain. However, because PTPRN is expressed in several other neuroendocrine tissues, other studies have shown that double knock-out of PTPRN and homologue PTPRN2 (IA2-β) causes female infertility due to a reduction in pituitary DCVs and subsequent lowering of serum luteinizing hormone levels, as well as anxiogenic behavior and learning deficits associated with a decrease of norepinephrine, dopamine and serotonin in the brain [65]. Since in our study we did not detect a significant increase in PTPRN2 during microarray analysis, and because our behavioral studies indicated that the hyperinsulinemic KK/HlJ mice with elevated PTPRN expression are well adapted for behavioral research studies and in some cases even superior to the C57BL/6 J strain [27], it is tempting to suggest that in certain types of behavior and learning tests PTPRN has a more pronounced effect than PTPRN2, as has indeed shown to be the case in studies by Cai and Notkins [64]. Furthermore, the biogenesis of DCVs are under the control of Chromogranin B (CHGB) [66], which also exhibited increased gene expression in the adrenal and pancreatic tissues of the KK/HlJ mice. Recent studies have shown that siRNA-targeted loss of CHGB in vitro impairs glucose-stimulated insulin secretion and reduces the density of insulin-containing granules [67]. Additionally, in the adrenal gland loss of CHGB in knockout mice was shown to reduce chromaffin granule abundance by approximately 35% which promoted deregulation of catecholamine release; whereas lentiviral over-expression led to a greater abundance of DCVs [68], suggesting a key role for CHGB and PTPRN in endocrine secretion. Increased expression of both PTPRN and CHGB genes in KK/HlJ mice may explain the higher levels of serum insulin and aldosterone that we observed in these mice.

In terms of sex-biased gene expression common to both tissues, we found as expected a number of well-characterized genes such as female-biased XIST and Y-chromosome markers Eif2s3y, Uty, DDX3Y and KDM5D [69]. One of the most interesting genes with male-biased expression was Neuronatin (Nnat), first identified as selectively expressed in newborn mice and involved in neurogesis, but more recently known to be a paternally expressed imprint gene [70] involved in a number of diverse functions including neuronal growth and brain development [71], synaptic plasticity [72], cellular stress response [73] and glucose-mediated insulin secretion [74]. Nnat has two splicing variant forms: the α form being composed of three exons which can be alternatively spliced to produce Nnat β. The expression of each isoform is tissue-specific and in some instances may be regulated by nutrient status, for example white adipose tissue Nnat is expressed at higher levels in mice fed a high-energy diet than in low-fat diet-fed mice [75]. Other studies have shown that whereas βcell-targeted knockout of Nnat in male mice fed a standard chow diet did not impair fasting blood glucose levels, βcell KO-Nnat+/−p mice fed a high-energy diet exhibited elevated fasting blood glucose together with glucose intolerance compared to wildtype C57BL/6 J mice, despite no apparent effect on body weight, feeding or energy expenditure [76]. In our study, all mice consumed the same standard chow diet, and the majority of the glucocentric differences that we observed were strain-related, although males from both strains had poorer insulin tolerance than the females. Given the diversity of functions attributed to Nnat, it would also be interesting to know the significance of the observed male-biased expression of Nnat in the adrenal tissue of these mice.

When we examined sex-biased differences in the pancreatic and adrenal tissues separately we found many interesting sex-bias differences in gene expression. This observation is in agreement with Yang et al who showed that in mice, sexually dimorphic genes are quite tissue-specific [77]. In the pancreas we found a subset of (MUPs) with clear sex-biased expression towards the males in both strains, as has been previously observed in other studies [78]. In the adrenal glands of these same mice however, no such bias in expression was observed. What was more interesting is that we found greater numbers of adrenal genes with sex-biased expression than pancreatic sex-biased DEGs. Moreover in the KK/HlJ adrenal glands there were larger numbers of female-biased genes compared to males, whereas in the C57BL/6 J adrenal glands, male-biased genes predominated; and the mirror opposite situation in the pancreata, as shown by our Venn diagram analysis. This is in agreement with previous studies of conserved mammalian sex-differences in gene expression, which have shown that across the body there are marked spatial differences in sex-bias, with some tissues have greater sex-biased genes than others. For example a recent study by Naqvi et al [79] found that mammalian adrenal and pituitary tissues have greater numbers of sex-biased DEGs compared to mammalian heart, liver, thyroid or brain. In humans, one study found that the heart and kidney express a number of DEGs with opposite trends in sex-bias. Genes from the RNA U1 family were found to be sex-biased towards the female in the heart, whereas in the kidneys the same genes were more abundantly expressed in males [80]. Other researchers have noted temporal differences in murine sex-biased expression in the sense that many genes can exhibit female-biased expression at one post-natal developmental time-point, and male-biased during another [81, 82], indicating that the transcription of sex-biased murine genes is regulated not only by genetic background, but by many influences, including epigenetic factors: hormonal, environmental, nutritional status, in addition to spatial and temporal variance.

In conclusion, we have carried out a comprehensive analysis of strain- and sex-biased differences in the expression of pancreatic and adrenal genes in male and female C57BL/6 J and KK/HlJ mice, as an extension of our previous work on the glucocentric, physiological and behavioral differences in these strains [27]. Our data may contribute to the understanding of differences in small-animal models for research into the pathogenesis of diabetes, obesity and associated disorders.

## Supplementary Information

**Additional file 1: Supplementary sTable 1.** List of genes used for RT-PCR analysis together with their Primer sequences.

## Data Availability

The datasets generated and analyzed during the current study are available in the NCBI GEO repository under the accession #s GSE141313 and GSE141310 at www.ncbi.nlm.nih.gov/geo/
