# Supplementary material for: Strain-based and sex-biased differences in adrenal and pancreatic gene expression between KK/HlJ and C57BL/6 J mice
Source: BMC Genomics. 2021 Mar 12;22:180. doi: 10.1186/s12864-021-07495-4 (PMC7953684; doi:10.1186/s12864-021-07495-4)
Supplement: Supplementary file 1 — Additional file 1: Supplementary sTable 1. List of genes used for RT-PCR analysis together with their Primer sequences. [file 12864_2021_7495_MOESM1_ESM.pdf]

Strain-based and Sex-biased Differences in Adrenal and Pancreatic Gene Expression between KK/HlJ and C57BL/6J mice.

Angela Inglis[1], Rosario Ubungen[1], Sarah Farooq[1], Princess Mata[1], Jennifer Thiam[1], Soad Saleh[1], Sherin Shibin[1], Futwan A. Al-Mohanna[1] & Kate S. Collison[1*].

[1]Department of Cell Biology, King Faisal Specialist Hospital & Research Centre,

PO BOX 3354, Riyadh 11211, Saudi Arabia.

Key words: Microarray, Strain, Gene Expression, Glucose Homeostasis, Insulin Tolerance Test, C57BL/6J, KK/HlJ, Sex-dependent.

***Corresponding author.**

Corresponding author and reprint requests:
Kate S. Collison PhD,
Department of Cell Biology,
King Faisal Specialist Hospital & Research Centre,
P. O. Box. 3354, Riyadh 11211,
Saudi Arabia.
Tel. () 96611-464-7272
Email. kate@kfshrc.edu.sa

Supplementary Table S1 List of gene specific primers for SYBR Green RT-PCR

Gene symbol	Gene name	Accession Number	Forward primer	Reverse primer
			CACCGAACTTACCACGACCT	ATGTCCTGTCTGCCAAAACC
AHSG	Alpha-2-HS-Glycoprotein	NM_001276449	GCAAGTAGCTTTGTTGGCCG	GCATGAGATTTGCCAGCGG
			CCCCGCTGGCAAATCTCAT	TCGTGGTAAGTTCGGTGGTC
CPS1	Carbamoyl-Phosphate Synthetase 1	NM_001080809	CTGGCTGGCTACCAAGAGTC	ATTGGGATCCACAAAATCCA
			GCCTCAACTGTACTGTCGCC	TGTCTTTGCCTTCACAGAAAGG
			TGGAGATCATGTGCTGCTTC	GCGACTATGAGCGTGAACAA
HMGCR	3-Hydroxy-3-Methylglutaryl-Coenzyme A Reductase	NM_008255	TGAGATCCGGAGGATCCAAG	CAGATCTTGTTGTTGCCGGTG
			TGAGATCCGGAGGATCCAAG	GATGCACCGCGTTATCGTCA
			TCAGAGGGAACTGGAGCACT	CGGAGACTGACCTCAAAAGC
APOB	Apolipoprotein B	NM_009693	CTACTTCCACCCACAGTCCCC	GGGCACATTGCTTTTTAGGGA
			TCAGAGGGAACTGGAGCACT	CGGAGACTGACCTCAAAAGC
			TACTTCCACCCACAGTCCCCT	CCTTAGAAGCCTTGGGCACAT
SERPINA1C	Serine (or Cysteine) Peptidase Inhibitor, Clade A, Member 1C	NM_009245	TTCCAACACCTCCTCCAAAC	AGGGTGTCCTTAGGCAGGAT
			CTAAAGAGGCCCAGAAGGTTAG	ATAAGGAACGGCTAGTAAGACTGT
VNN1	Vanin 1	NM_011704	CTACCAAGGTCTGGGAACCA	AGGGTGTCCTTAGGCAGGAT
			ACTTTCCTCGCGGCTGTTTA	CAATAATGTGCGCACCCTGC
			GTGACCCTGTCAGCCACTCT	GGTCTCCTTTCCTCCACCTC
GAL	Galanin	NM_010253	ACCGAGAGAGCCTTGATCCT	GGTTGTCAATGGCATGTGGG
			CCCACATGCCATTGACAACC	CCGGCCTCTTTAAGGTGCAA
			CTTGCACCTTAAAGAGGCCG	ATTGGCTTGAGGAGTTGGCA
			TAGGCCAGGCCATTCTAAGC	TCATTCCCTGGCTTCAGAGAC
Akr1c18	Aldo-Keto Reductase Family 1, Member C18	NM-134066	TCTCTGAAGCCAGGGAATGAGC	ACGGTTAAAGTTAGACACCCCG
			ATACTTCGAAGCTTTGGTCAACT	TAGGCAAAAGCTCATTCCCTGG
GPAM	Glycerol-3-Phosphate Acyltransferase, Mitochondrial	NM-008149	AGGCGCAGAGCCGAAGC	AAGTCCCAAACCATGTGCTGAC
			AGCAAGTCCTGCGCTATCAT	CTCGTGTGGGTGATTGTGAC
			TTTCCTGGGCGAGATTATTG	TGAATGGAATGAGGGCTTTC
SRD5A2	Steroid 5 Alpha-Reductase 2	NM_053188	TGCCTTCTGCATAGGGAACG	AAACAAGCCACCTTGTGGGAT
			CCACAAGGTGGCTTGTTTACG	GGGCTTTCCTAGATTTGGGGT
			TCAAAGCACACCAGACTTGC	ATGACCCTGCACCAGAAATC
FETUB	Fetuin Beta	NM_001083904	GTGGCTTACAGTGGGAAGACAT	AGTCAGGGCACGTTGTATGA
			TGACATTGTGCAGTGGCTCA	GATCCCATGTCTTCCCACTGT
			CGGGAACACCTGGAGAGGAT	CAGGCCCATTAGTCCCGTTG
APPL2	Adaptor Protein, Phosphotyrosine Interaction, PH Domain and Leucine	NM_145220	AACAAGACCGGACTGGTCAC	ATCGTCGGTCTTCACAATCC
			GGCTCCGAGCTAAGTCTCAA	AGGCACATTTCATTCTGTGCG
LY6D	Lymphocyte Antigen 6 Complex, Locus D	NM_010742	AGACAGCTCTGCTCGTCCTC	TTCAGAGGCTCCACTGAGGT
			AAAACCGTCACCTCAGTGGA	GACCAGCCTCTCGTTGCATA
Xdh	Xanthine Dehydrogenase	NM_011723	ATGGCAAAAAGGTGGTGGAGA	TCAAGCAGGCATTGACGGAA
			TCCAGCTAACGTCCAGCTTT	TGGCTTCTGAAGTGTCGATG
Ptprn	Protein Tyrosine Phosphatase, Receptor Type, N	NM_008985	ATC GCA TGG CCA AAG GAG TG	CCA AGG TGG CAG ATG GGT AG
Ddx3y	Dead (Asp-Glu-Ala-Asp) Box Polypeptide 3, Y-Linked	NM_012008	GGA TTT GGT GGA GGT GGC TA	AGG GCT ACA GGT TGT TGC TTA
			AGTGATCCTGCCAAGAGGAG	GAGGTTACAGCATCCAGCACT
HIST1H2BC	Histone Cluster 1, H2bc	NM_023422	AACGACATCTTCGAGCGCAT	TGTCAGGTCTGCTCCTCTTGG
			TCGTGAACGACATCTTCGAG	GTCAGGTCTGCTCCTCTTGG
			CTCTGGCTCTTGGGTTTCTG	GCTTCTGTTGACCCAGCTTC
NMNAT2	Nicotinamide Nucleotide Adenylyltransferase 2	NM_175460	CCCATCATGACCGAGACCAC	CCCTGGCTCTCTCGAACATC
			GATGTTCGAGAGAGCCAGGG	GTCGGAATTCTGGACAGCCA
			GATGTTCGAGAGAGCCAGGG	GTGGGCACATTGCTGTTCTG
Snord53	Small Nucleolar RNA, C/D Box 53	NR_028551	TGGTTTCGCGTCTGTCTGAG	AACAGCCAAGAGAAAGGTGTCA
			CCTCAGTGGCTGAGAAGACC	TTGCTCTTTGGCTTCTTGGT
SNCG	Synuclein, Gamma	NM_011430		GACCAAGGAGGGGGTTATGT
			CCAAGCAGGGAGTAACGGAG	GGTTCCAAGTCCTCCTTGCG
			GACCAAGCAGGGAGTAACGG	CTTCTCAGCCACTGAGGTGAC
			TACAAGCCTCGACAGACACG	GGTCAAATTGGGAAGAAGCA
SLC19A2	Solute Carrier Family 19 (Thiamine Transporter), Member 2	NM_001276455	TGAACGGCCTCAAGGAGGAG	CGCGTAGTTCACCACTTGGA
			TGAACGGCCTCAAGGAGGAG	CAAGGTTGAAACGGCCTCCA
			CTCCAGCCTGAAGTGGAGTC	GCATCTTGCAGATGGTCAGA
C7	Complement Component 7	NM_001243837	CCCAAGCATGCAGGTGACAA	ACTGAACGCCTTCGAGTCTG
			ACTGTGGGGGAGACAAGAGC	CCTTCGAGTCTGAGTCTTGGTA
			AGCCAGACAGTGGAGGAAGA	GAGCAGAATGTGGCTGATGA
Glycam1	Glycosylation Dependent Cell Adhesion Molecule 1	NM_001289587	ATGTCTTGGGAGGTGCAACCA	TGATGAAGGCACCACCTAGC
			AGCCCACAGATGCCATTCCA	CTGGCTCATACCAGCTGAGGT
			GGATCCAGAAGGTGATGGAA	AGAGCTGACAGCAACCCTGT
ARG2	Arginase Type II	NM_009705	TAGGGTAATCCCCTCCCTGC	GCTTCTTCTGTCCCCGAGAG
			TCCTTGCGTCCTGACGAGAT	GGTGGCATCCCAACCTGGAG
Snord47	Small Nucleolar RNA, C/D Box 47	NR_028543	GTGATGATTCTGCCA	CCTCAGAAATAAAATG
